# Airway mucins promote immunopathology in virus-exacerbated chronic obstructive pulmonary disease

**DOI:** 10.1172/JCI120901

**Published:** 2022-04-15

**Authors:** Aran Singanayagam, Joseph Footitt, Matthias Marczynski, Giorgia Radicioni, Michael T. Cross, Lydia J. Finney, Maria-Belen Trujillo-Torralbo, Maria Calderazzo, Jie Zhu, Julia Aniscenko, Thomas B. Clarke, Philip L. Molyneaux, Nathan W. Bartlett, Miriam F. Moffatt, William O. Cookson, Jadwiga Wedzicha, Christopher M. Evans, Richard C. Boucher, Mehmet Kesimer, Oliver Lieleg, Patrick Mallia, Sebastian L. Johnston

**Affiliations:** 1 National Heart and Lung Institute, Imperial College London, London, United Kingdom.; 2 Centre for Molecular Bacteriology and Infection, Imperial College London, London, United Kingdom.; 3 School of Engineering and Design, Department of Materials Engineering and; 4 Center for Protein Assemblies, Technical University of Munich, Munich, Germany.; 5 Marsico Lung Institute/Cystic Fibrosis and Pulmonary Research Center, University of North Carolina at Chapel Hill, Chapel Hill, North Carolina, USA.; 6 Division of Pulmonary Sciences and Critical Care Medicine, University of Colorado, Anschutz Medical Campus, Aurora, Colorado, USA.; 7 College of Health, Medicine and Wellbeing, Hunter Medical Research Institute and University of Newcastle, Newcastle, New South Wales, Australia.

**Keywords:** Pulmonology, COPD, Innate immunity

## Abstract

The respiratory tract surface is protected from inhaled pathogens by a secreted layer of mucus rich in mucin glycoproteins. Abnormal mucus accumulation is a cardinal feature of chronic respiratory diseases, but the relationship between mucus and pathogens during exacerbations is poorly understood. We identified elevations in airway mucin 5AC (MUC5AC) and MUC5B concentrations during spontaneous and experimentally induced chronic obstructive pulmonary disease (COPD) exacerbations. MUC5AC was more sensitive to changes in expression during exacerbation and was therefore more predictably associated with viral load, inflammation, symptom severity, decrements in lung function, and secondary bacterial infections. MUC5AC was functionally related to inflammation, as *Muc5ac*-deficient (*Muc5ac^–/–^*) mice had attenuated RV-induced (RV-induced) airway inflammation, and exogenous MUC5AC glycoprotein administration augmented inflammatory responses and increased the release of extracellular adenosine triphosphate (ATP) in mice and human airway epithelial cell cultures. Hydrolysis of ATP suppressed MUC5AC augmentation of RV-induced inflammation in mice. Therapeutic suppression of mucin production using an EGFR antagonist ameliorated immunopathology in a mouse COPD exacerbation model. The coordinated virus induction of MUC5AC and MUC5B expression suggests that non-Th2 mechanisms trigger mucin hypersecretion during exacerbations. Our data identified a proinflammatory role for MUC5AC during viral infection and suggest that MUC5AC inhibition may ameliorate COPD exacerbations.

## Graphical abstract

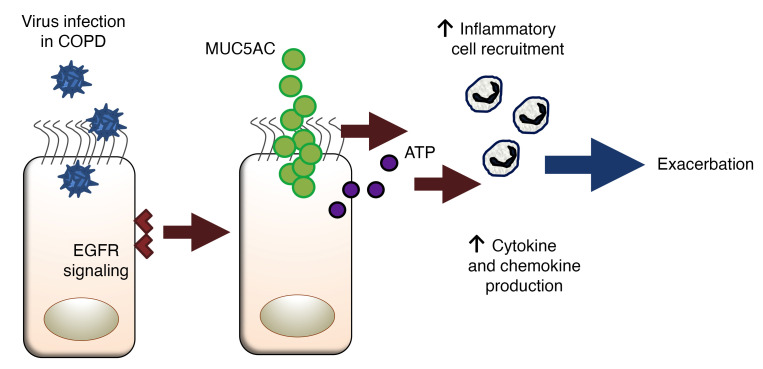

## Introduction

Chronic obstructive pulmonary disease (COPD) is an inflammatory airway disorder punctuated by acute exacerbations that are frequently precipitated by rhinoviruses (RVs) (1, 2). Exacerbations are the major cause of morbidity and mortality in patients with COPD (3, 4), and a greater understanding of the underlying pathophysiological mechanisms is needed to facilitate the development of new therapies to reduce the impact of these debilitating episodes. Respiratory mucosal surfaces are continuously exposed to inhaled pathogens, and a protective layer of secreted mucus that is rich in mucin glycoproteins acts as a first line of defense against infection. Abnormalities in mucus secretion, including increased expression of the major airway mucins MUC5AC and MUC5B, are a cardinal feature of inflammatory airway diseases such as COPD (5–7). The complexity of interactions between mucins and pathogens and how the perturbations in mucin expression that occur in COPD affect susceptibility to infection and subsequent exacerbations are poorly understood. Nor is it known whether inhibition of mucus production would be beneficial or harmful in the context of exacerbations.

Animal studies have identified critical roles for MUC5AC and MUC5B in antiviral and antibacterial host defense (8, 9), suggesting that therapies targeting mucin production may compromise the protective role of mucins during exacerbations. Conversely, mucin concentrations are higher in COPD patients who experience frequent exacerbations (7, 10), raising the speculation that mucins exert a causal role in exacerbation pathogenesis. Moreover, airway MUC5AC correlates with lung function decline in COPD (10), mediates airway hyperresponsiveness in an asthma model in mice (11), and is overexpressed in cases of fatal asthma (12), suggesting that overexpression of mucins can produce adverse pulmonary outcomes in addition to increasing the frequency and severity of exacerbations. These studies support an opposing view that targeted suppression of mucin production during exacerbations might provide desirable improvements in outcomes.

In the present study, we combined human clinical studies, animal models, and in vitro airway epithelial culture systems to investigate the role of abnormal mucin secretion in the pathogenesis of COPD exacerbations. First, to elucidate the broad signaling pathways that trigger increased mucin production during exacerbations, we measured the absolute concentrations of both MUC5AC and MUC5B mucins in respiratory secretions during spontaneous or experimentally induced exacerbations in individuals with COPD by immunologic and mass spectroscopic techniques. Second, we performed a series of studies in mice and in vitro human airway cultures, utilizing purified mucins, to explore MUC5AC-specific roles in COPD exacerbation pathogenesis.

## Results

### MUC5AC is induced during naturally occurring COPD exacerbations and is enhanced in individuals with frequent exacerbations.

**Figure 1:**
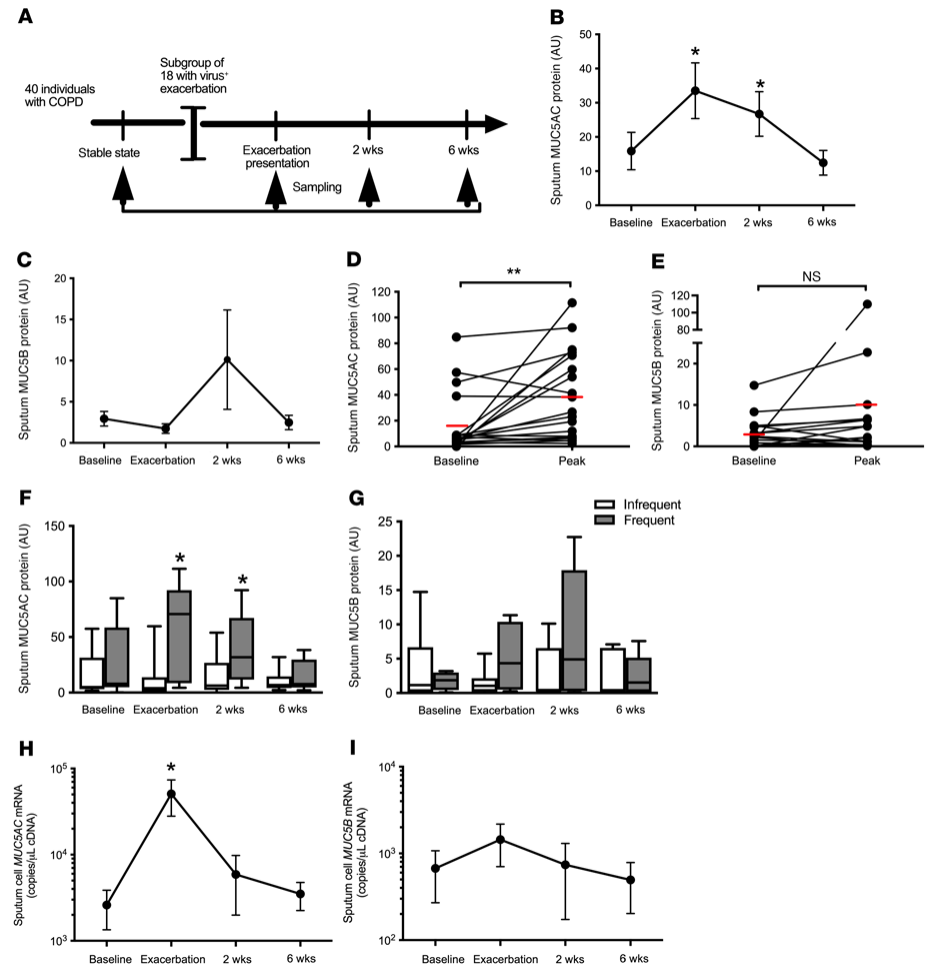
Airway mucin expression during naturally occurring, virus-induced COPD exacerbations. (**A**) A cohort of 40 patients with COPD was monitored prospectively. Sputum samples were taken during stable state (baseline), at presentation with an exacerbation associated with positive virus detection, and 2 and 6 weeks after exacerbation presentation. (**B**) Sputum MUC5AC and (**C**) MUC5B protein concentrations measured by ELISA. Baseline and peak (i.e., maximal concentrations of mucin detected during infection for each individual) concentrations of (**D**) MUC5AC and (**E**) MUC5B proteins during naturally occurring exacerbations. (**F**) Sputum MUC5AC and (**G**) MUC5B concentrations in individuals with frequent versus infrequent COPD exacerbations. (**H**) Sputum cell *MUC5AC* and (**I**) *MUC5B* mRNA expression. The red line in **D** and **E** indicates the mean concentration. In **F** and **G**, the box and whisker plots show the median (line within the box), the IQR (box), and the minimum-to-maximum values (whiskers). *P < 0.05 and **P < 0.01, by Mann-Whitney *U* test or Wilcoxon matched-pairs, signed-rank test.

We initially evaluated the expression of the major mucin glycoproteins MUC5AC and MUC5B in sputum samples obtained from patients with COPD during virus-associated, naturally occurring exacerbations in our community-based cohort study (Figure 1A). A total of 27 exacerbations were reported, of which 18 (67%) were associated with positive virus detection by PCR of sputum samples (RV *n =* 11, coronavirus *n =* 4, RSV *n =* 2, influenza *n =* 1), as previously reported (13). These 18 virus-positive exacerbation samples were used for analyses (see Table 1 for the clinical characteristics of the individuals who reported these 18 exacerbations). We observed significant increases from baseline in sputum MUC5AC protein concentrations at exacerbation presentations and at the 2-week point during exacerbations by immunologic approaches (Figure 1B). Although an approximately 2.5-fold increase in MUC5B was observed at 2 weeks, the response was variable and not statistically significant (Figure 1C). Peak exacerbation MUC5AC protein concentrations (i.e., maximal concentrations detected during exacerbation) were increased compared with baseline, with no significant increases detected for MUC5B (Figure 1, D and E). Patients who experienced frequent exacerbations (those with a history of ≥2 exacerbations in the preceding year) had increased sputum MUC5AC concentrations at exacerbation presentation and at 2 weeks during exacerbation compared with patients whose exacerbations were infrequent (0 or 1 exacerbations in the preceding year), with a similar trend observed for MUC5B that did not reach statistical significance (Figure 1, F and G). Given that ELISA-based quantification of sputum mucins may be influenced by proteases present in sputum samples (14), we measured mucin expression by quantitative PCR (qPCR) in RNA extracted from sputum cells from the same individuals. We observed that sputum *MUC5AC* mRNA expression was significantly increased at exacerbation onset, with no significant increase in *MUC5B* mRNA (Figure 1, H and I). These data provide evidence that measurements of MUC5AC more sensitively track COPD exacerbations than those for MUC5B.

### COPD is associated with augmented MUC5AC responses to experimental RV challenge.

**Figure 2:**
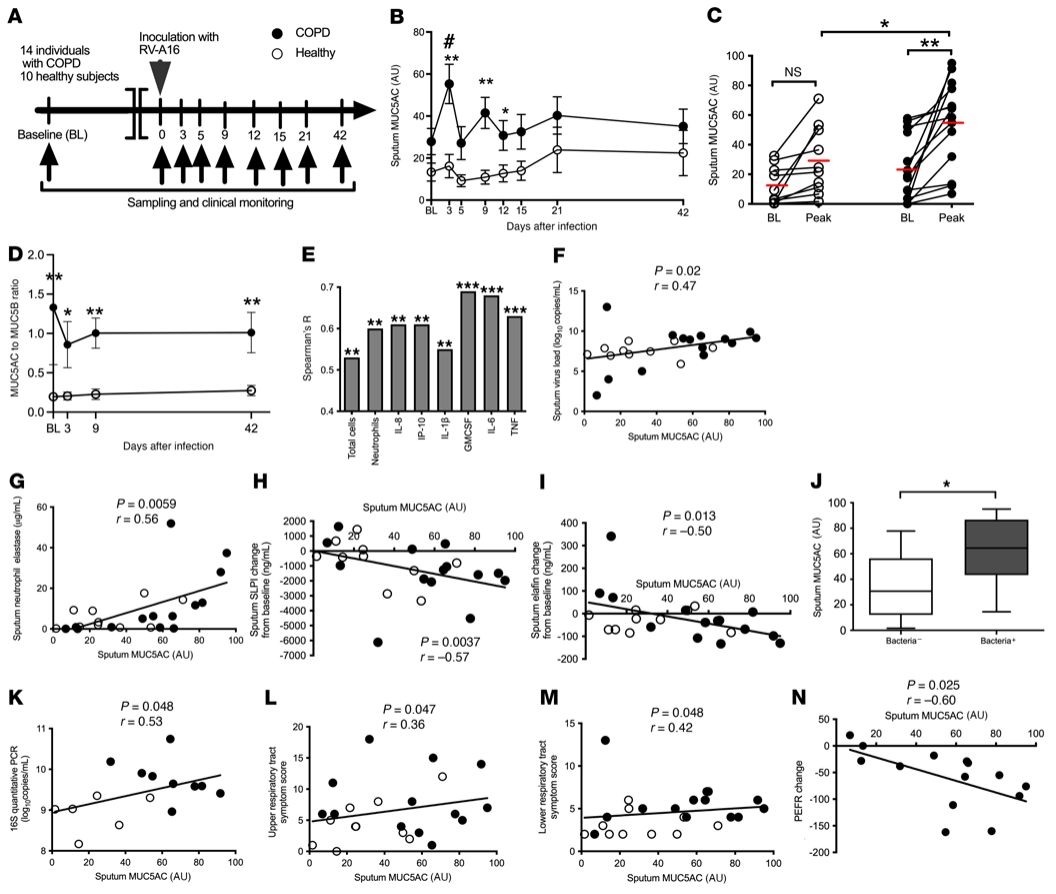
Airway MUC5AC expression and correlation with immunopathology during experimental RV infection. (**A**) Experimental outline. Fourteen patients with COPD and 10 healthy control volunteers underwent sampling at baseline and the indicated time points after RV-A16 infection. (**B**) Sputum MUC5AC concentrations in patients with COPD and healthy individuals, measured during RV-A16 infection. (**C**) Comparison of baseline (BL) and peak (i.e., maximal concentration detected during the infection for each individual) concentrations of MUC5AC in patients with COPD and healthy individuals. Individual data points are shown; red lines indicate the mean concentration. (**D**) Sputum MUC5AC to MUC5B ratio measured by mass spectrometry for 11 individuals with COPD and 10 healthy participants. (**E**) Correlation of peak sputum MUC5AC concentrations with inflammatory cell numbers and cytokine concentrations in sputum. Correlations between peak sputum MUC5AC levels and (**F**) sputum viral loads and (**G**) sputum neutrophil elastase. Change from baseline concentrations of (**H**) SLPI and (**I**) elafin. (**J**) Sputum MUC5AC concentrations in patients with COPD with positive (+ve) or negative (–ve) sputum bacterial cultures during experimental RV infection. Box and whisker plots in **J** show the median (line within the box), the IQR (box), and the minimum-to-maximum values (whiskers). (**K**) Correlation of peak sputum MUC5AC with bacterial loads as assessed by 16S qPCR. 16S qPCR was not measured for all patients because of a lack of sample availability, so only the data measured are shown. (**L**) Upper respiratory tract symptom scores. (**M**) Lower respiratory tract symptom scores. (**N**) PEFR change from baseline. (**B** and **D**) *P *<* 0.05 and **P < 0.01 (COPD vs. healthy); ^#^*P <* 0.05 (day 3 vs. baseline in patients with COPD). (**C**) *P < 0.05 and **P < 0.01, by Wilcoxon matched-pairs, signed-rank test for baseline versus peak values, and Mann Whitney *U* test for comparison of peak values between patients with COPD and healthy individuals. (**J**) *P < 0.05, by Mann Whitney *U* test. (**E**) **P < 0.01 and ***P < 0.001, by Spearman’s correlation analysis of pooled data on healthy volunteers and patients with COPD. (**F**–**I** and **K**–**N**) Nonparametric Spearman’s correlation analysis was performed on pooled data on healthy volunteers and patients with COPD.

We next used our previous human RV challenge studies (1, 15) to more accurately define the temporal dynamics of mucin expression during RV-induced COPD exacerbations (study is outlined in Figure 2A and Supplemental Figure 1A; supplemental material available online with this article; https://doi.org/10.1172/JCI120901DS1). The clinical characteristics of these individuals are described in Table 2. We observed a significant increase in sputum MUC5AC from baseline in RV-infected COPD patients on day 3 after infection, with no increase observed in healthy control individuals (Figure 2B). MUC5AC concentrations were increased in patients with COPD versus those in healthy controls on days 3, 9, and 12 after infection (Figure 2B). We noted a trend toward increased MUC5B on day 15 after infection in patients with COPD that did not reach statistical significance, and we observed no differences in MUC5B longitudinal responses between these 2 groups during infection (Supplemental Figure 1B). No significant differences in the magnitude of the MUC5B responses were noted between patients with COPD and healthy volunteers (Supplemental Figure 1B). MUC5AC peak concentrations were increased in patients with COPD compared with baseline, with no significant change observed in healthy individuals. Peak MUC5AC concentrations were higher in patients with COPD than in healthy individuals (Figure 2C). MUC5B peak concentrations were increased in both groups of volunteers when compared with baseline, with no significant difference observed between the 2 groups (Supplemental Figure 1C).

### Measurement of MUC5AC and MUC5B by mass spectroscopy before and during exacerbations.

To gain more direct insight into mucin-related inflammatory pathways during exacerbations, in a subset for which sufficient sample amounts were available (*n =* 11 patients with COPD, *n =* 10 healthy individuals, Supplemental Figure 2A), we performed stable isotope–labeled mass spectrometry to quantify the changes in MUC5AC and MUC5B concentrations during COPD exacerbations and to calculate the ratios of MUC5AC to MUC5B over time. Mass spectrometric measurements of MUC5AC and MUC5B during experimentally induced exacerbations mirrored those obtained by ELISA. We observed a similar induction of MUC5AC above baseline in patients with COPD on day 3 and an increase in concentrations of MUC5AC in patients with COPD versus healthy individuals on days 3 and 9 after infection (Supplemental Figure 2B). Consistent with our ELISA findings, measurement by mass spectrometry also showed trends toward increased MUC5B on day 9 compared with baseline that did not reach statistical significance, and no differences were detected between patients with COPD and healthy participants during infection (Supplemental Figure 2C). Notably, studies of type 2 asthma have reported that type 2 inflammation is associated with induction of *MUC5AC* and suppression of *MUC5B* mRNA expression (leading to an increased overall MUC5AC to MUC5B ratio >100) (16, 17). In contrast to type 2 high asthma (16), the ratio of MUC5AC to MUC5B protein observed in our study was approximately 0.8 to 1.0 (Figure 2D). We observed no significant increase in the ratios from baseline during infection in our experimentally infected individuals with COPD (Figure 2D), suggesting a coordinated upregulation of both MUC5AC and MUC5B during RV infection in the individuals with COPD. The ratio of MUC5AC to MUC5B was increased in individuals with COPD versus healthy individuals at all time points during infection, as previously reported (refs. 7, 10, and Figure 2D).

### RV-induced MUC5AC is related to the severity of COPD exacerbation.

Having characterized the expression of mucins during exacerbation, and noting that MUC5AC induction occurred on day 3, a time point that preceded the peak airway inflammation that occurs between days 9 and 15 after infection (1, 15), we next sought to assess associations between mucin responses and inflammatory responses to infection in our human experimental challenge model. MUC5AC was selected to test for a correlation, because its greater dynamic range provided more sensitivity. We observed highly significant positive correlations between sputum MUC5AC concentrations and cellular airway inflammation (total cell counts and neutrophil counts, Figure 2E) and between MUC5AC concentrations and concentrations of soluble mediators of inflammation, including CXCL8 (aka IL-8), CXCL10 (aka IP-10), IL-1, GM-CSF, IL-6, and TNF (Figure 2E). Since virus replication acts as a driver for airway inflammation (1, 15), we also examined relationships between sputum MUC5AC concentrations and viral load and found a positive correlation between these parameters (Figure 2F).

We have previously reported that RV infection induces secondary bacterial infection in individuals with COPD through neutrophil elastase cleavage of the antimicrobial peptides (AMPs) secretory leucocyte proteinase inhibitor (SLPI) and elafin to promote bacterial growth (18). We found that sputum MUC5AC concentrations correlated positively with sputum neutrophil elastase concentrations (Figure 2G), and negatively with the change from baseline in sputum SLPI and elafin concentrations, during infection (Figure 2, H and I). These findings suggest that increased MUC5AC concentrations during infection are associated with greater induction of neutrophil elastase and, consequently, increased degradation of these AMPs, as previously reported (18). Patients with COPD with positive bacterial cultures during RV infection also had higher concentrations of sputum MUC5AC during infection (Figure 2J), and their sputum MUC5AC concentrations correlated with bacterial loads determined by 16S qPCR (Figure 2K). To confirm the observations in the RV challenge model, we performed similar analyses in the naturally occurring COPD exacerbation study and found that sputum MUC5AC concentrations during virus-induced exacerbations also correlated with bacterial loads measured 2 weeks after exacerbation onset, a time point we have previously shown to represent the peak of secondary bacterial infections (ref. 18 and Supplemental Figure 3A). We conducted similar analyses for MUC5B, which did not significantly correlate with any inflammatory marker except GM-CSF (Supplemental Figure 1D). Similarly, there were no significant correlations of MUC5B concentrations with viral load (Supplemental Figure 1E), changes from baseline in AMP concentrations (Supplemental Figure 1, F and G), or bacterial loads during experimental (Supplemental Figure 1H) or naturally occurring COPD exacerbation (Supplemental Figure 3B).

Proinflammatory cytokines and cellular airway inflammation are believed to contribute to the duration and severity of RV-induced exacerbations (19, 20). Secondary bacterial infection is also associated with greater exacerbation severity in RV-induced COPD exacerbations (18). Having observed associations between concentrations of MUC5AC and airway inflammation, viral load, and secondary bacterial infections, we next determined whether airway mucins during exacerbations were related to clinical outcomes during exacerbation. We observed that sputum MUC5AC concentrations correlated with both upper and lower respiratory tract symptom scores (Figure 2, L and M), with no significant correlations detected for MUC5B (Supplemental Figure 1, I and J). Since chronic mucus hypersecretion and elevated sputum MUC5AC are associated with a long-term decline in forced expiratory volume in 1 second (FEV_1_) (10, 21), we also hypothesized that the induction of MUC5AC during RV infection would be related to acute lung function changes during COPD exacerbation. In experimental RV-induced COPD exacerbations, sputum MUC5AC concentrations correlated negatively with the maximal fall from baseline in peak expiratory flow during exacerbation (Figure 2N), with no such correlation detected for MUC5B (Supplemental Figure 1K).

To confirm these findings, we additionally measured MUC5AC concentrations in an alternative sample type, bronchoalveolar lavage (BAL), as participants in the experimental infection study also underwent bronchoscopy at baseline and on day 7 after RV infection. BAL MUC5AC protein concentrations were similarly increased in patients with COPD versus healthy nonsmokers, with no difference observed for MUC5B (Supplemental Figure 4, A and B).

Collectively, these observations confirmed that MUC5AC and MUC5B are probably both induced by RV infection. However, given the lower basal concentrations of MUC5AC and its reactivity to RV infection, MUC5AC fold-change concentrations were more sensitive to RV-induced mucin changes than to MUC5B fold changes. Accordingly, we observed that changes in MUC5AC concentrations correlated with inflammation, viral load, secondary bacterial infections, and clinical measures of exacerbation severity.

### Muc5ac^–/–^ mice have attenuated inflammatory responses to RV infection.

**Figure 3:**
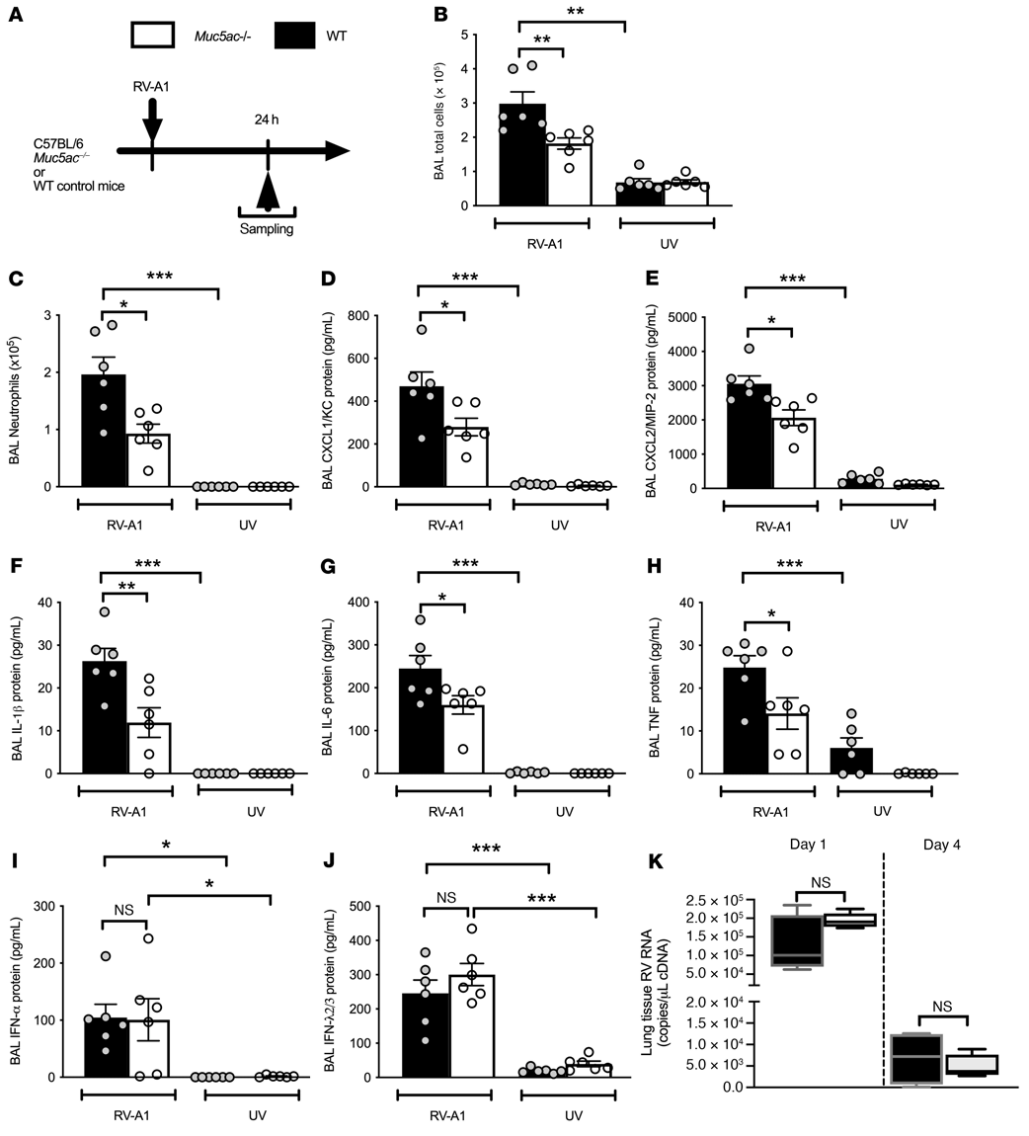
*Muc5ac^–/–^* mice have attenuated inflammatory responses to RV infection. (**A**) Experimental outline. *Muc5ac^–/–^* and WT control C57BL/6 mice were i.n. infected with RV-A1 or UV-inactivated RV-A1. (**B**) BAL total cell count and (**C**) BAL neutrophil count on day 1 after infection. BAL concentrations of (**D**) CXCL1/KC, (**E**) CXCL2/MIP-2, (**F**) IL-1β, (**G**) IL-6, (**H**) TNF, (**I**) IFN-α, and (**J**) IFN-λ2/3 on day 1 after infection. (**K**) Lung tissue RV RNA copies. Box and whisker plots in **K** show the median (line within the box), the IQR range (box), and the minimum to maximum values (whiskers). Data in **B**–**J** are expressed as the mean ± SEM of 6 mice per treatment group and are representative of 2 independent experiments. *P < 0.05, **P < 0.01, and ***P < 0.001, by 1-way ANOVA with Bonferroni’s post test.

We next sought to understand whether the elevated MUC5AC concentration had functional consequences over and above simple increases in total mucin concentrations, using mouse models in which cause-and-effect relationships could be assessed (Figure 3A). We have previously reported that RV-A1 infection induces airway Muc5ac and Muc5b secretion in mice (22). We initially confirmed RV-A1 induction of total cellular and neutrophilic airway inflammation as well as induction of 2 neutrophil chemokines and 3 proinflammatory cytokines in WT mice (Figure 3, B–H). These responses required replicating virus, as we observed a highly significant (all *P <* 0.001) induction with live RV-A1 that was completely absent with UV-inactivated RV-A1 (Figure 3, B–H). Mice with gene-targeted deletion of *Muc5ac* (*Muc5ac^–/–^*) had attenuated cellular airway inflammation (BAL total cell counts and neutrophil numbers; Figure 3, B and C) and reduced concentrations of the neutrophil chemokines CXCL1 (aka KC) and CXCL2 (aka MIP-2) following RV infection compared with WT controls (Figure 3, D and E). BAL concentrations of the proinflammatory cytokines IL-1, IL-6, and TNF were also reduced in RV-infected *Muc5ac^–/–^* mice compared with WT controls (Figure 3, F–H). The proinflammatory effects of MUC5AC were not mediated via interference with antiviral immune responses or virus proliferation, as *Muc5ac^–/–^* mice and WT control mice had similar concentrations of IFN-α and IFN-λ2/3 protein in BAL and no difference in lung viral loads (Figure 3, I–K). These data indicated that MUC5AC is functionally related to increased airway inflammatory responses during RV infection.

### Exogenous MUC5AC protein augments RV-induced airway inflammation and increases bacterial loads in mice.

**Figure 4:**
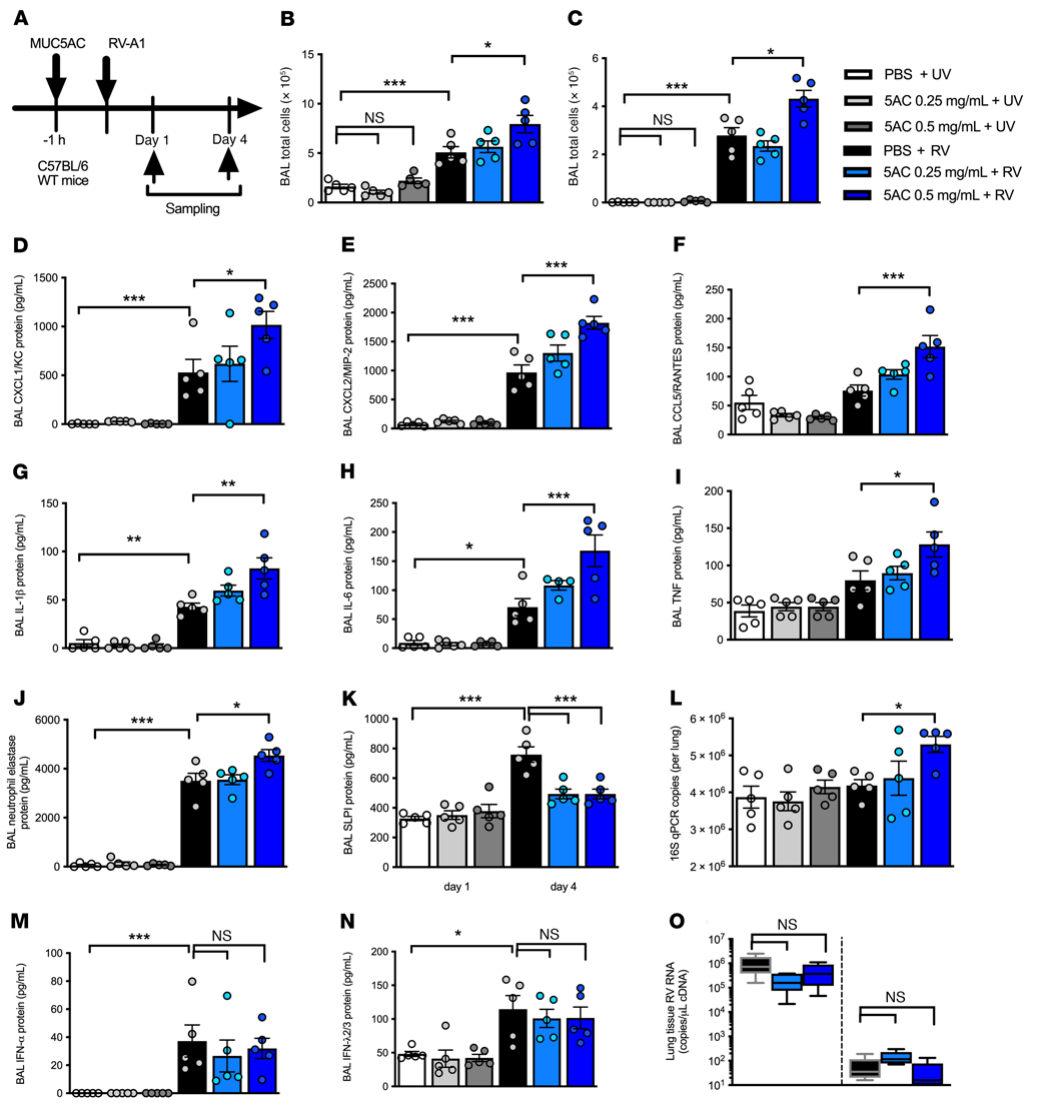
Exogenous MUC5AC augments airway inflammation and bacterial loads in RV-infected mice. (**A**) Experimental outline. C57BL/6 WT mice were treated i.n. with purified MUC5AC protein and additionally i.n. infected with RV-A1 or UV-inactivated RV-A1. (**B**) BAL total cell counts and (**C**) BAL neutrophil counts on day 1 after infection. BAL concentrations of (**D**) CXCL1/KC, (**E**) CXCL2/MIP-2, (**F**) CCL5/RANTES, (**G**) IL-1β, (**H**) IL-6, (**I**) TNF, (**J**) neutrophil elastase, and (**K**) SLPI on day 1 after infection. (**L**) Lung 16S bacterial loads on day 4 after infection. (**M**) IFN-α and (**N**) IFN-λ2/3 protein concentrations in BAL on day 1 after infection. (**O**) Lung tissue RV RNA copies. Box and whisker plots in **O** show the median (line within the box), the IQR (box), and the minimum to maximum values (whiskers). Data in **B**–**N** are expressed as the mean ± SEM of 5 mice per treatment group and are representative of 2 independent experiments. *P < 0.05, **P < 0.01, and ***P < 0.001, by 1-way ANOVA with Bonferroni’s post test.

To further evaluate the role of MUC5AC in RV-induced inflammation and exacerbation severity, we evaluated the in vivo effects of exogenous MUC5AC glycoprotein in the mouse model of RV infection (Figure 4A). Exogenous MUC5AC administration alone had no effect on BAL inflammatory cell numbers (Figure 4, B and C) or on proinflammatory chemokine or cytokine concentrations (Figure 4, D–I). However, MUC5AC administration (0.5 mg/mL) enhanced RV-induced airway inflammation, including an increase in total BAL cell and neutrophil numbers (Figure 4, B and C) and proinflammatory chemokines (CXCL1/KC, CXCL2/MIP-2, and CCL5 [aka RANTES] and cytokines (IL-1, IL-6, and TNF) (Figure 4, D–I). Conversely, administration of MUC5B protein or a control polymer solution of agarose/dextran at the same concentration (0.5 mg/mL) had no effect on inflammation, either alone or when administered in combination with RV infection in mice (Supplemental Figure 5).

Consistent with our hypothesis that MUC5AC drives neutrophil elastase–mediated cleavage of AMPs and subsequently increases bacterial loads, we also observed that exogenous MUC5AC increased RV induction of neutrophil elastase (Figure 4J), suppressed RV induction of SLPI (Figure 4K), and augmented lung bacterial loads during viral infection (Figure 4L). Similar to what we observed with knockout mice, exogenous MUC5AC also had no effect on the induction of IFN-α or IFN-λ2/3 or on lung viral loads in RV-infected mice (Figure 4, M–O).

### Exogenous MUC5AC protein augments proinflammatory responses to RV infection in human airway epithelial cells.

Mucus coats airway epithelia, which are a major source of proinflammatory cytokines during RV infection. Having observed that exogenous MUC5AC augmented RV-induced inflammation in mice, we next examined the effect of the administration of MUC5AC protein on RV-infected bronchial epithelial cells (BECs) in vitro (Supplemental Figure 6A). In BEAS2B BECs, exogenous MUC5AC had no effect when administered with UV-inactivated virus. In contrast, MUC5AC dose-dependently enhanced induction of the proinflammatory cytokines CXCL8 (aka IL-8) and IL-6 when accompanied by live RV (Supplemental Figure 6, B and C).

### MUC5AC exerts proinflammatory effects via extracellular adenosine triphosphate.

We next investigated how MUC5AC amplifies inflammation during RV infection. Given our prior observations that *Muc5ac* deletion was antiinflammatory and that administration of exogenous MUC5AC protein to mice and BECs had broad proinflammatory effects, we reasoned that Muc5ac could mediate its effects via a danger signal that promoted inflammatory mediator release and cell recruitment. Extracellular adenosine triphosphate (ATP) (23), a well-recognized promoter of inflammation (24), is virus inducible (25). We therefore hypothesized that MUC5AC exerts its proinflammatory effects during RV infection via induction of extracellular ATP release. Importantly, we found that exogenous MUC5AC protein administration augmented ATP release in RV-infected BECs (Supplemental Figure 6D)

**Figure 5:**
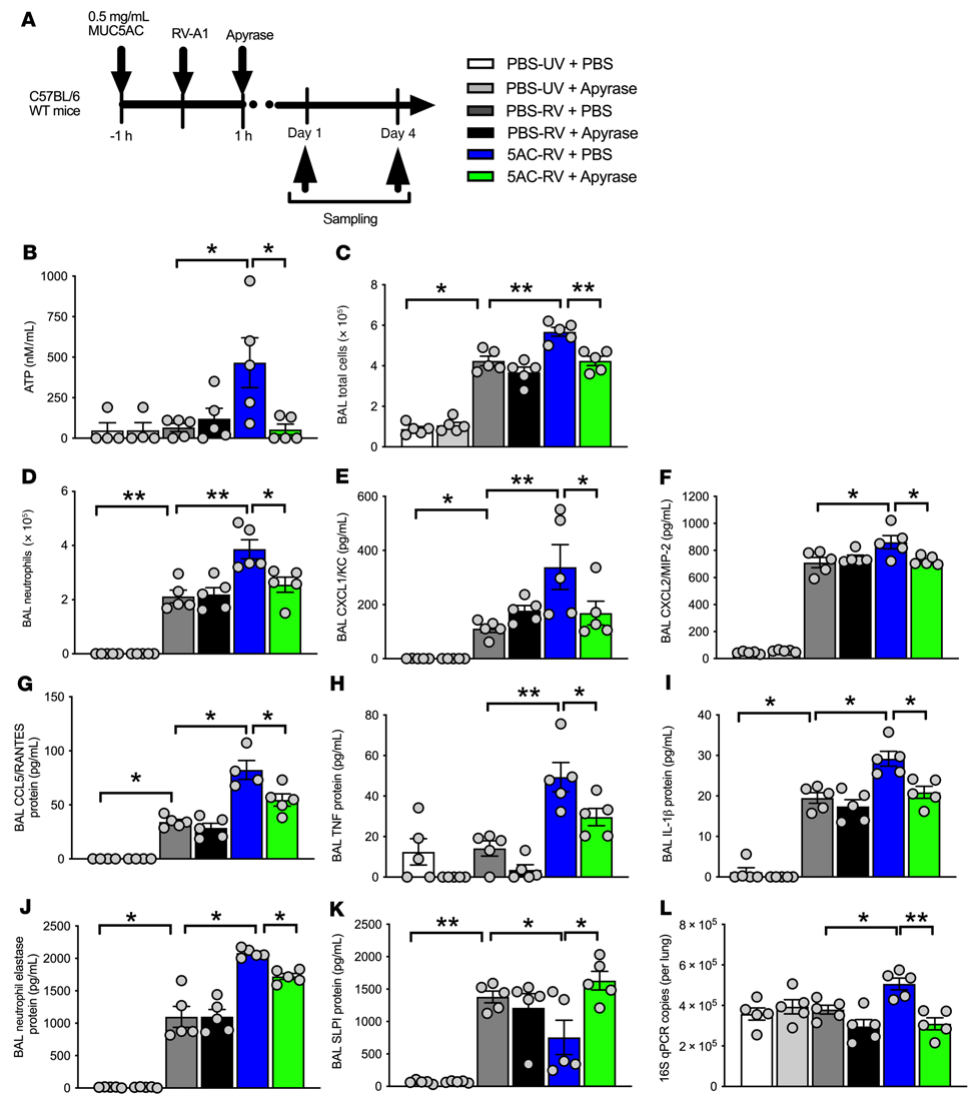
Neutralization of airway ATP inhibits augmentation of inflammation and bacterial loads by exogenous MUC5AC. (**A**) Experimental outline. C57BL/6 WT mice were treated i.n. with purified MUC5AC protein or PBS control, infected with RV-A1 or UV-inactivated RV-A1, and additionally treated with i.n. apyrase or vehicle control (PBS). (**B**) BAL ATP concentrations on day 1 after infection. (**C**) BAL total cell counts and (**D**) BAL neutrophil counts on day 1 after infection. BAL concentrations of (**E**) CXCL1/KC, (**F**) CXCL2/MIP-2, (**G**) CCL5/RANTES, (**H**) TNF, (**I**) IL-1β, (**J**) neutrophil elastase, and (**K**) SLPI on day 1 after infection. (**L**) Lung 16S bacterial loads on day 4 after infection. All data are expressed as the mean ± SEM of 5 mice per treatment group and are representative of 2 independent experiments. *P < 0.05 and **P < 0.01, by 1-way ANOVA with Bonferroni’s post test.

We next examined the effect of ATP neutralization on in vivo inflammatory responses using the ATP-hydrolyzing enzyme apyrase in exogenous MUC5AC–treated, RV-infected mice (Figure 5A). Exogenous MUC5AC protein administration augmented BAL concentrations of ATP in RV-infected mice (Figure 5B), an effect that was not observed after administration of exogenous MUC5B or agarose/dextran solution (Supplemental Figure 5I). MUC5AC augmentation of ATP was abrogated by the instillation of apyrase (Figure 5B). Apyrase administration also suppressed exogenous MUC5AC–mediated increases in airway inflammation during RV infection, including BAL total cell neutrophil numbers (Figure 5, C and D) as well as expression of the inflammatory chemokines CXCL1/KC, CXCL2/MIP-2, and CCL5/RANTES (Figure 5, E–G) and the proinflammatory cytokines TNF and IL-1 (Figure 5, H and I). Apyrase also prevented the exogenous MUC5AC–mediated increases in neutrophil elastase, reversed the MUC5AC-mediated suppression of SLPI, and suppressed MUC5AC-mediated increased bacterial loads in RV-infected mice (Figure 5, J–L). Apyrase-mediated reversal of the proinflammatory effects of MUC5AC did not occur via interference with antiviral immune responses or virus replication, as we observed no effects of apyrase on RV induction of IFN-α, IFN-λ2/3 (Supplemental Figure 7, A–C), or lung viral loads (Supplemental Figure 7D). These data suggest that MUC5AC may exert its proinflammatory effects during RV infection through induction of the release of extracellular ATP.

### Therapeutic inhibition of MUC5AC induction by RV protects against exacerbation in a mouse model of COPD-like disease.

**Figure 6:**
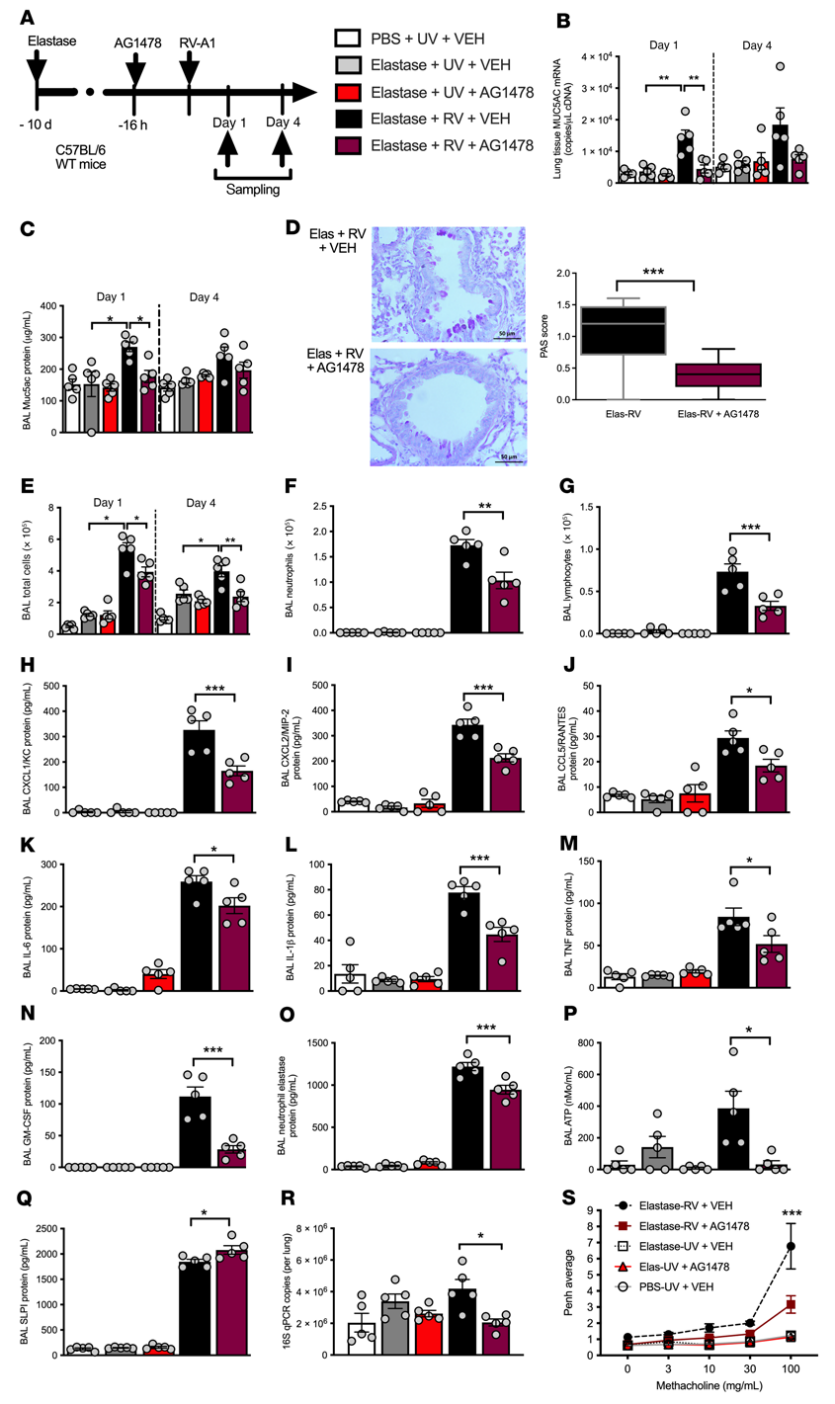
EGFR inhibition inhibits MUC5AC expression and attenuates airway inflammation, bacterial loads, and airway hyperreactivity in a mouse model of virus-exacerbated COPD. (**A**) Experimental outline. C57BL/6 WT mice were treated i.n. with elastase or PBS control and additionally treated i.p. with 50 mg/kg of the EGFR inhibitor AG1478, prior to challenge with RV-A1 or UV-inactivated RV-A1. (**B**) Lung *Muc5ac* mRNA expression and (**C**) BAL MUC5AC protein concentration. (**D**) Periodic acid–Schiff–stained (PAS-stained) lung sections on day 4 after infection, with scoring for PAS-positive, mucus-producing cells. Scale bars: 50 μm. Original magnification, ×400. (**E**) BAL total cell counts and (**F**) BAL neutrophil counts on day 1 after infection. (**G**) BAL lymphocytes on day 4 after infection. BAL concentrations of (**H**) CXCL1/KC, (**I**) CXCL2/MIP-2, (**J**) CCL5/RANTES, (**K**) IL-6, (**L**) IL-1β, (**M**) TNF, (**N**) GM-CSF, (**O**) neutrophil elastase, (**P**) ATP, and (**Q**) SLPI, on day 1 after infection. (**R**) Lung 16S bacterial loads on day 4 after infection. (**S**) Airway hyperresponsiveness (enhanced pause [Penh]) to methacholine challenge on day 1 after infection. In **S**, the comparison shown is for elastase-RV plus vehicle versus the elastase-RV plus AG1478 group. All data are expressed as the mean ± SEM of 5 mice per treatment group and are representative of 2 independent experiments. *P < 0.05, **P < 0.01, and ***P < 0.001, by 1-way ANOVA (**B**–**R**) and 2-way ANOVA (**S**) with Bonferroni’s post test. Elas, elastase; VEH, vehicle.

Having confirmed the functional importance of MUC5AC in RV-induced airway inflammation, we next hypothesized that inhibiting RV induction of MUC5AC would have beneficial effects during RV-induced COPD exacerbations. RVs induce MUC5AC expression through EGFR signaling (26). We therefore assessed the effect of EGFR inhibition in a mouse model of elastase-induced emphysema combined with RV infection, in which many features of human COPD exacerbation, including mucus hypersecretion, are recapitulated (27). Administration of the EGFR inhibitor AG1478 prior to RV infection in elastase-treated mice (Figure 6A) suppressed RV induction of lung *Muc5ac* mRNA and BAL Muc5ac protein (Figure 6, B and C), and suppressed airway epithelial cell mucus staining in lung sections (Figure 6D). AG1478 had no effect on lung mRNA expression or BAL protein concentrations of Muc5b protein (Supplemental Figure 8, A–C). Administration of AG1478 to elastase-treated mice also suppressed RV induction of BAL total cells, neutrophilic and lymphocytic cellular airway inflammation (Figure 6, E–G), as well as chemokines CXCL1/KC, CXCL2/MIP-2, and CCL5/RANTES (Figure 6, H–J), proinflammatory cytokines IL-1, IL-6, TNF, and GM-CSF (Figure 6, K–N), neutrophil elastase (Figure 6O), and extracellular ATP (Figure 6P). AG1478 administration to elastase-treated, RV-infected mice also enhanced BAL SLPI concentrations and reduced pulmonary bacterial loads (Figure 6, Q and R). Elastase- and AG1478-treated, RV-infected mice had reduced airway hyperresponsiveness (AHR) to methacholine challenge compared with elastase- and vehicle-treated, RV infected controls (Figure 6S).

These antiinflammatory effects of AG1478 were not mediated via interference with antiviral immune responses or virus control, as we also observed no effect of AG1478 on RV induction of IFN-α, IFN-λ2/3 (Supplemental Figure 8, D and E), or lung viral loads. (Supplemental Figure 8F).

## Discussion

The mechanisms underlying the virus-induced exacerbations in COPD, and the factors that drive inflammation and the clinical severity of these episodes are poorly understood. Here, we provide insights into the roles that the mucin glycoproteins MUC5AC and MUC5B play in the biology of these episodes. Using a combination of human and mouse models of RV infection in COPD, we demonstrate that MUC5AC and MUC5B are increased during exacerbations, that MUC5AC has better characteristics than MUC5B as a biomarker of COPD exacerbations, and that virus-induced MUC5AC may have a role in driving COPD exacerbation severity.

Our clinical data demonstrated that MUC5AC and MUC5B were expressed during virus-induced COPD exacerbations and increased compared with levels in virus-infected healthy individuals. The lower basal concentration and reactivity of MUC5AC to viral infection made MUC5AC a more sensitive biomarker of airway inflammatory responses to viral infection. However, the constancy of the MUC5AC to MUC5B ratio during infection, coupled with the absolute increases in MUC5B, indicated that RV induced both MUC5AC and MUC5B expression. This coordinate upregulation of MUC5AC and MUC5B has been previously reported with RV in COPD in vitro (28). Importantly, from a mucin gene regulatory perspective, the coordinate upregulation of MUC5AC and MUC5B suggests that classic Th2 signaling, which raises MUC5AC expression but suppresses MUC5B expression, is not dominant in RV responses in COPD. Consistent with previous reports, this pattern suggests that EGFR ligands, IL-1α/-1β, IL-6, and/or TNF-α contribute to this regulatory response (29).

Capitalizing on the greater sensitivity of MUC5AC as a mucin biomarker, we investigated correlations between MUC5AC and other cardinal features of COPD exacerbation. Positive correlations were detected between MUC5AC concentrations and multiple indices of the inflammation that characterize COPD exacerbations (19, 20). We also investigated relationships between changes in MUC5AC concentrations and exacerbation severity. Positive correlations with viral loads, symptom scores, and acute lung function decline were detected. MUC5AC, as one of the major glycoprotein components of airway mucus, exhibited predictable correlations with lower respiratory symptom scores, which included an assessment of sputum production. The observed correlation between MUC5AC concentrations and peak expiratory flow rate (PEFR) decline may be explained by the likelihood that greater mucus production contributes directly to airway obstruction during exacerbations. Our findings in this acute human infection model are in keeping with clinical studies that have reported associations between chronic mucus hypersecretion/sputum MUC5AC concentrations with airflow limitation and FEV_1_ decline (10, 21, 30) and an animal study showing that genetic deletion of *Muc5ac* reduced mucus occlusion and improved lung function in models of allergic airway inflammation (11).

We have previously reported that experimental RV infection in patients with COPD is associated with an increased frequency of secondary bacterial infection compared with healthy individuals. One potential mechanism relates to virus-induced, neutrophil elastase–mediated cleavage of the AMPs SLPI and elafin, as reported in our prior studies (18). The positive correlation between sputum MUC5AC and neutrophil numbers, neutrophil elastase concentrations, and secondary bacterial infections, and the negative correlation with SLPI and elafin concentrations observed in the current study, raise speculation that increased MUC5AC could be a further component of this mechanism, but further studies in which components of this pathway are manipulated in mouse models will be required to confirm this definitively. Furthermore, increased MUC5AC may contribute to secondary bacterial infections by other mechanisms. For example, Siegel et al. previously demonstrated reduced secondary pneumococcal growth following influenza infection in *Muc5ac^–/–^* mice (31). This effect was attributable to reduced provision of mucin-derived nutrients for bacterial growth in *Muc5ac^–/–^* mice, indicating that MUC5AC may be an important promoter of secondary bacterial infection. In addition, the increased MUC5AC concentrations, coupled with increased MUC5B concentrations, are predicted to increase mucus concentration–dependent osmotic pressures sufficiently to produce cessation of mucus transport, mucus accumulation, and mucus plugging (32). Static mucus is a preferred site for secondary bacterial infection in other diseases, e.g., cystic fibrosis (CF), and probably serves a similar role in virus-induced COPD exacerbation (33).

Recent findings from the SPIROMICS (SubPopulations and InteRmediate Outcome Measures In COPD Study) cohort have shown that total mucin concentrations in sputum during stable periods are higher in patients with COPD who experienced 2 or more exacerbations compared with those with zero exacerbations (7, 10). Previous studies have also reported that chronic mucus hypersecretion and sputum MUC5AC are associated with an increased frequency of exacerbations (10, 34, 35) and mortality risk related to pulmonary infection (36). Here, we found that patients who had frequent exacerbations also had increased sputum mucin concentrations during these exacerbations. The underlying mechanisms involved in the frequent exacerbation phenotype are poorly characterized, but our data hint at the idea that these patients may have exaggerated mucin production at baseline and in response to infection.

The human experimental challenge model facilitates sequential sampling at precisely defined time points during the course of infection and allows evaluation of temporal relationships between variables. We observed that MUC5AC induction peaked on day 3 in the time course of these RV-induced COPD exacerbations, preceding airway cellular inflammation, which peaked between days 9 and 15 after infection (1, 15). One possible explanation for this sequence is that mucus plugging consequent to mucus hypersecretion is proinflammatory, as suggested in CF and COPD-like mouse models (37, 38). Given the strong positive correlations observed between MUC5AC and inflammatory parameters in the current study, a second possibility is that MUC5AC induced by RV might contribute directly to enhanced airway inflammation during exacerbations. Accordingly, we carried out complementary gain- and loss-of-function experiments in mice to directly investigate the functional role of MUC5AC during RV infection. Mice with gene-targeted deletion of *Muc5ac* (*Muc5ac^–/–^*) had attenuated airway inflammation following RV infection, effects similar to those reported in models of ventilator-induced lung injury in the same knockout strain (39). However, a study by Cho et al. reported the opposite finding, as *Muc5ac^–/–^* mice had increased pulmonary injury in response to respiratory syncytial virus (RSV) infection (40). The reasons for the differences between our findings and these are unclear, but it is possible that roles for mucins may differ according to virus type.

We additionally observed that exogenous MUC5AC protein administration augmented RV-induced proinflammatory responses in mice, which also suggests that MUC5AC is related to enhanced airway inflammation during viral infection. Notably, these effects were not observed with administration of MUC5B protein or a control polymer of an agarose/dextran solution. Exogenous MUC5AC also enhanced RV-induced neutrophil elastase concentrations, suppressed SLPI production, and increased pulmonary bacterial loads, further suggesting a possible role for MUC5AC in inhibiting antibacterial responses during viral infection. Similar effects were observed in cultured BECs, in which direct MUC5AC administration augmented the release of proinflammatory cytokines in response to RV. However, it should be noted that these experiments were conducted within a submerged culture system, and these findings require future confirmation in more complex in vitro systems (e.g., air-liquid interface differentiated epithelial cells).

A previous study by Ehre et al. evaluated responses to influenza infection in a transgenic *Muc5ac-*overexpressing mouse line and reported reduced viral titers and attenuated neutrophilic inflammation in this model (9). This finding contrasts with our observations using exogenous MUC5AC administration, which had no effect on viral loads and enhanced rather than reduced virus-induced inflammation. The transgenic strain used by Ehre et al. was associated with an approximately 18-fold increase in airway MUC5AC expression (9). This magnitude of augmentation greatly exceeds the increases observed in our human analyses, in which an approximately 2-fold increase in MUC5AC concentrations was observed in patients with COPD versus healthy individuals, and an approximately 2- to 3-fold increase in mucin was observed from baseline to exacerbation. The concentrations of exogenous MUC5AC protein administered in our animal experiments (0.25–0.5 mg/mL) were approximately 1.5- to 3.5-fold higher than the baseline MUC5AC protein concentrations measured in the mouse airway (~150 μg/mL) and are, therefore, likely to mimic human exacerbations. Further, the effect of MUC5AC on reducing viral loads in the MUC5AC-transgenic strain reported by Ehre et al. was likely virus specific (9). The authors reported that MUC5AC bound to the influenza virus receptor via α2,3-linked sialic acids in transgenic animals. This finding is consistent with a mechanism of influenza protection in vivo in the *Muc5ac*-transgenic strain via impairment of virus binding to receptors on the bronchial epithelium, leading to a reduction in virus proliferation and, consequently, less inflammation.

It is important to note that we used a nonhuman purified gastric mucin protein preparation for our experiments that did not fully recapitulate the complex biochemical and biophysical properties of mucus lining airway surfaces. We confirmed that the mucin preparation used was free of endotoxin and DNA, but it is important to note that we cannot exclude the possibility that other mucin-bound proteins were present in the solution that may have contributed to the observed effects, as shown previously for purified MUC5B preparations (41). Previous immunofluorescence evaluation of the purified gastric mucin preparation we used indicated the presence of some MUC6 (42), although subsequent mass spectrometric analysis confirmed that only low levels were present (43). However, the mucin preparation used in our studies has been shown to be purer and more structurally and functionally representative of in vivo mucins than commercially available preparations (43). Future validation of our findings using administration of human or mouse MUC5AC in similar mouse models is warranted, although extraction and purification of mucins from these sources is technically challenging and was not feasible for the current studies.

Our data suggest a potential mechanism for amplification of RV-induced airway inflammation by MUC5AC through the release of ATP, a danger signal that is produced during infection and contributes to nucleotide receptor–mediated inflammatory responses (44). The role of ATP as a possible driver of airway inflammation in stable asthma and COPD is well recognized (45, 46). Our infection model findings that metabolism of pulmonary ATP release by apyrase abrogated MUC5AC enhancement of inflammation and secondary bacterial infection identify a potential mechanism through which RV-induced mucin production enhances the severity of COPD exacerbations. Whether ATP is directly induced by MUC5AC or if this occurs via another protein that interacts with this mucin remains unclear. Elucidation of the precise molecular mechanisms involved requires further investigation.

Inflammation in the lung is characterized by a number of positive feedback cycles. For example, our observations suggest that MUC5AC directly augmented RV-induced airway inflammation. However, a number of previous studies have also demonstrated that inflammatory mediators, e.g., neutrophil elastase and IL-1, directly induce MUC5AC and MUC5B expression in the lung epithelium (47). We therefore speculate that mucin-induced airway inflammation might trigger further production of mucins, leading to a vicious cycle that contributes to enhanced airway inflammation and mucus hypersecretion to drive exacerbation severity in COPD. Further studies using exogenous MUC5AC protein administration in *Muc5ac*-deficient mice will be required to formally interrogate this. We hypothesized that early targeting of mucins, and perhaps early inflammation, during RV infection might beneficially interrupt these cycles. Accordingly, we evaluated the effect of upstream inhibition of RV induction of MUC5AC using the EGFR inhibitor AG1478 in a COPD exacerbation mouse model. AG1478 suppressed RV-induced Muc5ac concentrations and simultaneously reduced airway inflammation, proinflammatory cytokines and chemokines, neutrophil elastase, and AHR, effects that may be directly related to, or independent of, Muc5ac attenuation. AG1478 also enhanced SLPI production and reduced bacterial loads in RV-infected, elastase-treated mice. Importantly, AG1478 had no impact on antiviral responses or on virus proliferation, indicating that therapies aimed at inhibiting MUC5AC production would not be expected to adversely affect antiviral host defense.

Jing et al. recently reported in COPD airway epithelial cell cultures that administration of an EGFR inhibitor had no effect on RV induction of *MUC5AC* and concluded that EGFR does not play a role in promoting virus-induced mucin expression in COPD (28). However, in their study, the authors measured mucin expression solely at a late time point (15 days) after RV infection and did not evaluate the effects of the treatment on mucin expression at earlier time points or the effects on production of relevant inflammatory mediators. The data from our human challenge model indicate that peak RV induction of MUC5AC occurred at a much earlier time point (3 days), and previous studies have shown that EGFR inhibition can attenuate the early induction of MUC5AC in RV-stimulated airway epithelial cell cultures (26, 48). Our data in a mouse model of COPD-like disease support the assertion that the early production of MUC5AC in response to RV infection occurs through EGFR and is thus amenable to therapeutic inhibition. However, it should be noted that EGFR inhibition during RV infection may have pleiotropic effects beyond the suppression of MUC5AC production, and we cannot conclusively determine whether the suppression of inflammation we observed was directly related to MUC5AC inhibition or some other mechanism. A previous clinical trial evaluating chronic therapy (4 weeks) with an inhaled EGFR inhibitor (BIBW 2948) in stable COPD reported a dose-dependent inhibition of EGFR activation with an associated reduction in epithelial mucin stores at higher doses (49). However, this drug was poorly tolerated by participants with notable adverse effects including FEV_1_ decline and liver function abnormalities being reported. Systemic EGFR antagonists are now commonly used in lung adenocarcinoma therapy (50), and shorter-course therapy at the onset of exacerbation may be more tolerable. In the current study, more profound effects on Muc5ac expression and associated immunopathology were observed in mouse models of virus-exacerbated rather than stable COPD. Our data in mice now provide justification for human studies to evaluate the role of repurposing these agents for use in COPD exacerbations to inhibit mucin production that could theoretically suppress airway inflammation, reduce secondary bacterial infection, and diminish exacerbation severity.

There were 2 limitations in this study with respect to mucin quantitation. First, there is the relative sensitivity of the ELISA-based measurement for MUC5AC versus MUC5B. Mucins were quantified in our study using ELISA-based assays in historically collected sputum samples within a separated sputum plug aliquot. Sputum plugs from people with severe asthma have previously been shown to contain more MUC5AC than MUC5B (51), which may have contributed to the increased detection of MUC5AC versus MUC5B in our studies. The immunologic assays also exhibited varying sensitivities for MUC5AC (high) versus MUC5B (low). However, the mass spectrometric analyses, coupled with other assays, confirmed that MUC5AC to MUC5B ratios were increased (~1.2) at baseline in patients with COPD compared with healthy controls (~0.2) and remained at similar ratios during exacerbations, consistent with coordinate upregulation of MUC5AC and MUC5B in individuals with resting or exacerbating COPD. Second, there are the mucin comparisons across study groups, i.e., COPD versus controls. There is evidence that proteases within sputum samples interfere with immunodetection of mucins (14). Some studies have attempted to address this issue by addition of protease inhibitors shortly after sputum collection (6, 52). We have previously reported, in samples from this study, that neutrophil elastase is increased in individuals with COPD compared with healthy individuals following virus challenge (18). Given that we observed higher resting concentrations and induction of MUC5AC and MUC5B protein in COPD sputum compared with that of healthy individuals, despite increased concentrations of proteases in the patients with COPD, it is possible that the difference between the 2 study groups could be even more substantial than was observed in our study. The primary aim of the human component of our study was to study how mucin concentrations change during virus-induced exacerbations. Our study was unique, as longitudinal samples were available from individual patients during exacerbation, who underwent mucin measurements to provide a consistent data set. It should be noted, however, that the mechanisms identified in the current study are focussed on virally driven exacerbations and may not be broadly applicable to other etiological causes of these episodes (e.g., primary bacterial infection, pollutants)

In conclusion, MUC5AC and MUC5B were both induced during viral infections in COPD. MUC5AC exhibited a greater dynamic range and may prove to be a superior biomarker for relating components of virus-induced disease pathogenesis to exacerbation severity. Finally, it is possible that virus-induced MUC5AC itself plays a central role in driving airway inflammation and exacerbation severity in COPD. Future development or repurposing of therapies that specifically target mucin upregulation, and perhaps MUC5AC specifically, could lead to improved clinical outcomes.

## Methods

### Experimental RV infection studies

Analysis was performed on biobanked samples from participants recruited to 2 experimental RV infection studies that have been published previously (1, 15, 18). Samples from 2 groups were included in this analysis: patients with COPD (Global Initiative for Obstructive Lung Disease [GOLD] stage II, not using regular inhaled therapy) and healthy nonsmokers. Baseline samples of induced sputum and BAL, collected and processed as previously described (1), were obtained when the participants had been free of COPD exacerbation/respiratory tract infection for at least 6 weeks, approximately 14 days prior to infection. The participants were inoculated with RV-A16 with samples collected on subsequent post-infection visits as described previously (1, 15). Sputum was induced using 4% saline administered by a DeVilbiss UltraNeb 99 ultrasonic nebulizer until an adequate sputum sample was obtained, and the sample was processed within 2  hours of induction. Sputum plugs were selected from saliva by macroscopic inspection of the sample. An aliquot was selected and stored unprocessed at –80°C. The remaining sample was weighed, and 0.1% DTT added. The mixture was then agitated and centrifuged, and the supernatant was stored at –80°C. Sputum from 14 patients with COPD and 10 nonsmoking healthy control individuals had complete remaining sample availability for the full time course from baseline to day 42 and were used for the present studies. Bronchoscopies were performed according to the British Thoracic Society Guidelines for research bronchoscopies (53). BAL was performed by instilling sterile, normal saline into the left upper lobe bronchus (30 mL aliquots up to a total of 240 mL). The collected fluid was filtered and centrifuged, and the supernatant was stored at –80°C. Daily diary cards of upper and lower respiratory tract symptom scores and PEFR measurements were recorded as previously described (1).

### The St. Mary’s Hospital naturally occurring COPD exacerbation cohort

Studies were performed using biobanked samples from a cohort of 40 patients with COPD recruited to a longitudinal study investigating the pathogenesis of naturally occurring exacerbations that was carried out at St. Mary’s Hospital London between June 2011 and December 2013, as previously reported (54). All participants had a clinical diagnosis of COPD that was confirmed with spirometry. Participants with all grades of COPD severity were recruited to the study, and all treatments were permitted.

All participants, when clinically stable, had an initial visit at baseline for clinical assessment, PEFR measurement, and clinical sample collection including sputum, taken using the same methodology as described above for the experimental infection studies. Participants reported to the study team when they developed symptoms of an upper respiratory tract infection or an increase in any of the symptoms of dyspnea, cough, and sputum volume or purulence, and an exacerbation was defined using the East London cohort criteria (4). The participants were seen within 48 hours of the onset of their symptoms for collection of samples, and repeat visits were scheduled for 2 and 6 weeks after the initial exacerbation visit, with clinical assessment, lung function measurement, and sputum induction repeated at these subsequent time points. Viruses were detected in sputum by PCR, as described previously (1).

### Mouse models

Female mice (aged 6–8 weeks) on a C57BL/6 background, (Charles River Laboratories) were used for all WT animal studies. *Muc5ac^–/–^* mice were generated on a C57BL/6 background at the University of Colorado, as previously described (11). Mice were housed in individually ventilated cages under specific pathogen–free conditions.

For the exogenous MUC5AC protein administration model, mice were i.n. infected under light isoflurane anesthesia with 50 μL of the minor group RV-A1 (5 × 10^6^ fifty-percent tissue culture infectious dose [TCID_50_]) or UV-inactivated RV-A1 control and additionally treated i.n. with 50 μL purified porcine MUC5AC protein at 0.25 and 0.5 mg/mL concentrations, 0.5 mg/mL purified MUC5B protein, and 0.5 mg/mL control polymer solution of purified agarose/dextran solution or PBS control, 1 hour prior to infection. Mucin proteins were prepared as previously described (55) and confirmed to be endotoxin free using a Limulus Amebocyte Lysate (LAL) assay kit (Lonza). Full details on the mucin protein purification and preparation are provided in the Supplemental Methods. In separate experiments, mice received 4 U/mL apyrase (MilliporeSigma), as previously described (46), in combination with exogenous MUC5AC and RV, 1 hour after infection.

For evaluation of the EGFR inhibitor in a COPD exacerbation model, as previously reported (27), mice were treated i.n. under light isoflurane anesthesia with 1.2 units of porcine pancreatic elastase (Merck) to induce COPD-like changes. Ten days after elastase administration, as previously described (56), mice were treated i.p. with 50 mg/kg of the EGFR inhibitor AG1478 (MilliporeSigma), 16 hours prior to i.n. infection with 50 μL RV-A1 (2.5 × 10^6^ TCID_50_) or the UV-inactivated RV-A1 control.

### Viral infection and treatment of BEAS-2B cells with MUC5AC protein

BEAS2B BECs (European Collection of Authenticated Cell Cultures [ECACC]) were cultured to 80% confluence in 12-well plates. Cells were subsequently stimulated with 0.2 mL RV-A1 (MOI = 2) or UV-inactivated RV-A1 control, as previously described (22), for 1 hour with shaking. Viruses were then removed and replaced with 1 mL media containing purified porcine MUC5AC at 0.1, 0.5, and 1 mg/mL concentrations (prepared as described above) followed by incubation for 8 hours at 37°C. Full details on the mucin protein purification and preparation are provided in the Supplemental Methods.

### Measurement of cytokines, mucin, and ATP concentrations

Cytokine protein concentrations in mouse BAL, human sputum supernatants, or cell supernatants from in vitro experiments were assayed using commercial DuoSet ELISA kits (R&D Systems). The MesoScale Discovery (MSD) platform was used to measure the inflammatory mediators IL-1, IL-6, TNF, CXCL8/IL-8, CXCL10/IP-10, and GM-CSF in sputum supernatants according to the manufacturer’s instructions, as published previously (15). Protein concentrations of human SLPI, elafin, neutrophil elastase, and all mouse BAL proteins were assayed using commercial ELISA assay kits (R&D Systems), as previously described (18, 22). MUC5AC and MUC5B proteins in previously stored human sputum plugs or human or mouse BAL were measured after adhesion to a 96-well plate by allowing the samples to evaporate at 37°C overnight, using in-house assays as previously described (22). For measurement of MUC5AC, the detection antibody used was biotinylated anti-MUC5AC (clone 45M1, Thermo Fisher Scientific, catalog 12639866) at 400 ng/mL. For the MUC5B assay, the detection antibody used was mouse anti-MUC5B (clone EH-Muc5Ba), as previously described (57). Bound anti-MUC5B antibody was detected with peroxidase-conjugated goat anti–mouse IgG (MilliporeSigma, catalog A4416). Mucins were quantified against the following standards: purified porcine MUC5AC, as previously described (55), and recombinant MUC5B protein (Caltag Medsystems).

Labeled mass spectrometry was performed to further quantify the absolute concentrations of MUC5AC and MUC5B mucins in sputum, as previously described (7, 10).

To measure ATP concentrations in mouse BAL supernatants, the ATPLite assay (PerkinElmer) was used according to the manufacturer’s instructions, but with the cell lysis step omitted, as previously described (46).

### RNA extraction, cDNA synthesis, and qPCR

For human studies, total RNA was extracted from sputum cells (RNeasy Kit, QIAGEN), and 2 μg RNA was used for cDNA synthesis (Omniscript RT Kit, QIAGEN). For mouse models, total RNA was extracted from the right upper lobe of mouse lung and placed in RNAlater (Thermo Fisher Scientific), prior to RNA extraction and cDNA synthesis. qPCR was carried out using previously described specific primers and probes for human and mouse *MUC5AC* and *MUC5B* and RV RNA copy numbers (22, 26, 58) and normalized to 18S rRNA.

### DNA extraction and bacterial 16S qPCR

DNA extraction from human sputum and mouse lung was performed using the FastDNA Spin Kit for Soil (MP Biomedicals), following the manufacturer’s instructions. Bead-beating was performed for 2 cycles of 30 seconds at 6800 rpm (Precellys, Bertin Technologies). 16S bacterial loads were measured using qPCR, as previously described (59).

### Statistics

Data from the human COPD studies were analyzed using the Wilcoxon matched-pairs, signed-rank test or the Mann-Whitney *U* test. Correlations between data sets were examined using Spearman’s rank correlation coefficient. For mouse experiments, the animals were studied in group sizes of 5 mice, and the data shown are representative of at least 2 independent experiments. Data were analyzed using 1- or 2-way ANOVA, with significant differences between groups assessed by Bonferroni’s multiple-comparison test. All statistical analyses were performed using GraphPad Prism 9 (GraphPad Software). Differences were considered significant when the *P* value was less than 0.05.

### Study approval

#### Experimental RV studies.

The study protocol was approved by St. Mary’s NHS Trust Research Ethics Committee (study nos. 00/BA/459E and 07/H0712/138).

#### St. Mary’s Hospital naturally occurring COPD exacerbation cohort.

The study protocol was approved by the East London Research Ethics Committee (protocol 11/LO/0229).

#### Animal studies.

AAll experiments were carried out under animal project licences PPL 70/7234 and P07D80C24, approved by the Animals in Science Regulation Unit, UK Home Office, London.

## Author contributions

AS, JF, PM, and SLJ conceived the study and designed the experiments. AS, JF, MM, GR, MTC, LJF, MC, MBTT, JA, PLM, OL, PM, and SLJ conducted the experiments. AS, PM, RCB, and MK analyzed the data. OL provided reagents. All authors contributed to the writing of the manuscript. CME, JW, JZ, NWB, and TBC conducted experiments. MFM and WOC conducted experiments and provided reagents.

## Supplementary Material

Supplemental data

## Figures and Tables

**Table 1:**
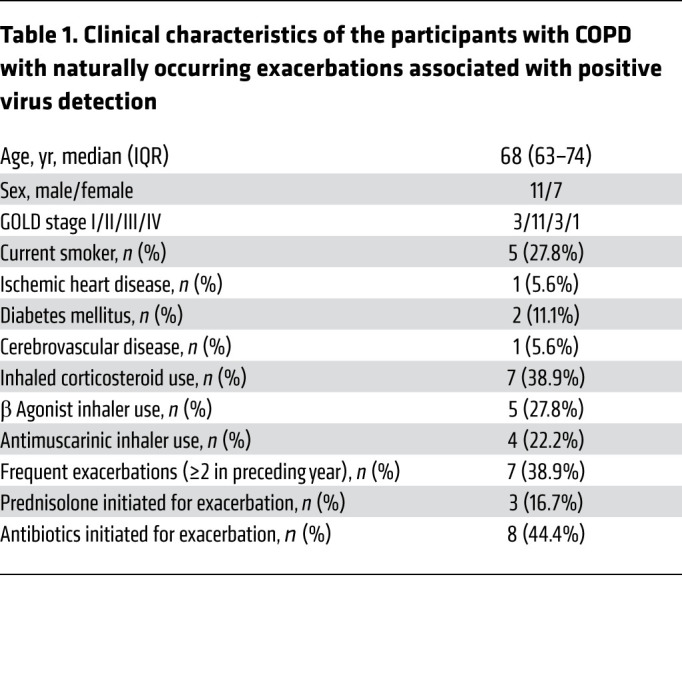
Clinical characteristics of the participants with COPD with naturally occurring exacerbations associated with positive virus detection

**Table 2:**
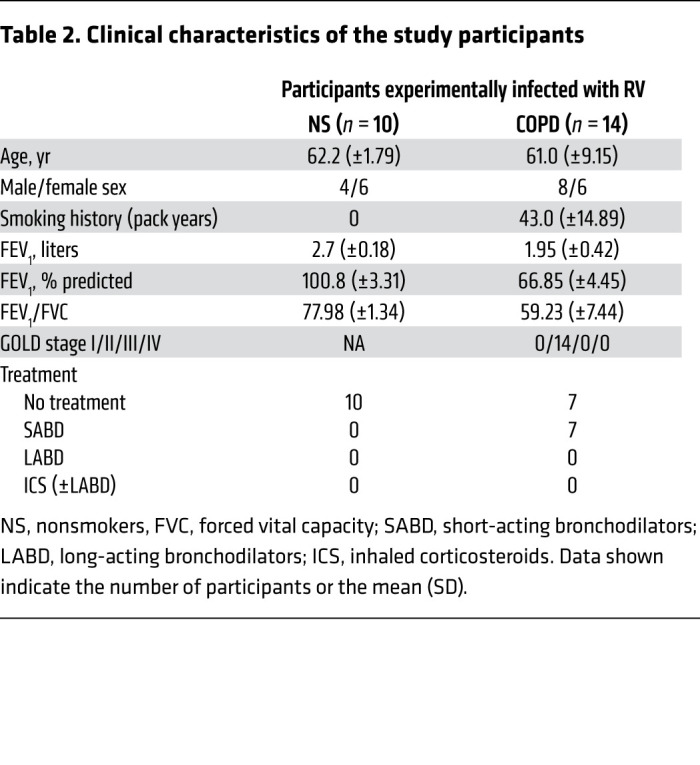
Clinical characteristics of the study participants

## Version 1 03/03/2022

In-Press Preview

## Version 2 04/15/2022

Electronic publication
