# Supplementary material for: Airway mucins promote immunopathology in virus-exacerbated chronic obstructive pulmonary disease
Source: J Clin Invest. 2022 Apr 15;132(8):e120901. doi: 10.1172/JCI120901 (PMC9012283; doi:10.1172/JCI120901)
Supplement: Supplemental data [file jci-132-120901-s035.pdf]

Supplementary methods

Purification of porcine gastric mucin

Previous studies revealed that the properties of commercially available mucins differ from key properties of native mucin systems as they, e.g. the lack of the ability to reduce friction [1-3] or lead to cytotoxic effects [4]. These differences are attributed to the harsh conditions during the commercial purification process [5]. As a consequence, here, the purification of porcine gastric MUC5AC is performed manually from fresh pig stomachs. An optimization of the original purification protocol from Celli *et.al* [6] was described in detail previously [3]. In brief, crude mucus was obtained by gently scraping pig stomachs after rinsing them with tap water. The mucus was diluted 5-fold in 10 mM sodium phosphate buffer (pH 7.0, supplemented with 170 mM NaCl) and homogenized by stirring at 4 °C overnight. Cellular debris was removed via several centrifugation steps (8.300 x g for 30 min at 4 °C and 15.000 x g for 45 min at 4 °C) and a final ultracentrifugation step (150.000 x g for 1 h at 4 °C). Afterwards, the supernatant was separated chromatographically by size-exclusion chromatography (ÄKTA purifier system, GE Healthcare, equipped with a XK50/100 column packed with Sepharose 6FF), and the fractions containing the mucin glycoproteins were identified via PAS (periodic acid/Schiff's base) staining and pooled. After a subsequent dialysis step against ultra-pure water via cross-flow filtration, the mucin-solution was concentrated, lyophilized and stored at -80 °C.

Repurification of commercial bovine salivary mucin MUC5B

Lyophilized bovine salivary mucin MUC5B powder was purchased from Merck (499643, Darmstadt, Germany) and dissolved in 10 mM sodium phosphate buffer (pH 7.0, supplemented with 1 M NaCl) at a concentration of 1 mg mL^{-1} and the solution was rotated on a rotating incubator for 1 h at 4 °C. Further purification of salivary mucin MUC5B was conducted according to the protocol used for purifying gastric mucin MUC5AC as described above.

Enzymatic removal of mucin-associated DNA

Since the gastric mucosa also contains DNA [7], enzymatic removal of potential mucin-associated DNA was performed to prevent any DNA-related inflammation reactions. All following steps were carried out under a sterile hood. Lyophilized mucin purified from porcine gastric mucosa was treated with UV-light on ice for 1 hour. Subsequently, it was dissolved in 50 mM Tris-HCl buffer (sterile filtered, pH 7.5, supplemented with 10 mM $MgCl_2$) at a concentration of 1 mg/mL and kept on ice. DNAse I from bovine pancreas (D5025, Sigma Aldrich, St. Louis, MO, USA) was dissolved in the same buffer at a concentration of 1 mg/mL. 50 µL of the DNAse solution were added per milligram of dissolved mucin. Afterwards, the mucin/DNAse solution was incubated at 37 °C for 18 hours in a tube shaker at 200 rpm. The treated mucin was then chromatographically separated from the enzyme and residual DNA fragments via size exclusion chromatography, subsequently desalted by means of cross-flow filtration and lyophilized (see *Purification of porcine gastric mucin* section above). The DNAse treated mucin was stored in lyophilized form at -80 °C.

Visualization of mucin-associated DNA

Successful removal of DNA from MUC5AC was verified by electrophoretic separation of the mucin samples and subsequent visualization of associated DNA molecules via the DNA-binding dye SYBR Green I (S9430, Sigma Aldrich, St. Louis, MO, USA). Therefore, 40 µg of the mucin samples were dissolved in 10 mM sodium phosphate buffer (pH 7.0) at a concentration of 1 % (w/v), and mixed with 2x Laemmli sample buffer (0.125 M Tris, 4 % (w/v) SDS, 20 % (v/v) glycerol, and 10 % (v/v) 2-mercaptoethanol) in a 1:1 ratio. The reducing agent 2-

mercaptoethanol was added to ensure that mucins are in their monomeric state. The samples were loaded on a polyacrylamide gel (Mini-Protean TGX Gels 4-20%, #45-1093, BioRad, Hercules, CA, USA) and separated (60 V for 10 min and 120 V until the dye front reaches the end of the gel). The gel was stained with SYBR Green I afterwards (diluted 1:10.000 in TBE buffer) at room temperature under gentle shaking for 30 min. The DNA stain was visualized with a Gel Doc EZ Gel Documentation System (Biorad) with epi-illumination at a wavelength of 254 nm (Supplementary Fig.7). The absence of a fluorescence signal in the DNase treated sample indicates successful removal of mucin-associated DNA.

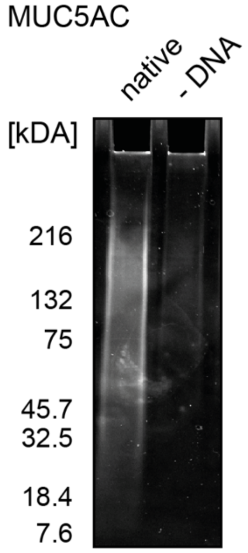

Supplementary Figure 9: SDS-PAGE of native and DNAse-treated porcine gastric MUC5AC. *DNA was removed from purified porcine gastric mucins by enzymatic treatment with DNAse I from bovine pancreas. Mucin samples were separated via gel-electrophoresis and subsequently stained with a SYBR Green nucleic acid gel stain to visualize associated DNA molecules (indicated by a white signal).*

References

1. Lee S. Characterization of Lubricity of Mucins at Polymeric Surfaces for Biomedical Applications. World Academy of Science, Engineering and Technology, International Journal of Medical, Health, Biomedical, Bioengineering and Pharmaceutical Engineering 2013;7:145-50.
2. Lee S, Muller M, Rezwan K, Spencer ND. Porcine gastric mucin (PGM) at the water/poly(dimethylsiloxane) (PDMS) interface: influence of pH and ionic strength on its conformation, adsorption, and aqueous lubrication properties. Langmuir : the ACS journal of surfaces and colloids 2005;21:8344-53.
3. Schömig VJ, Käsdorf BT, Scholz C, Bidmon K, Lieleg O, Berensmeier S. An optimized purification process for porcine gastric mucin with preservation of its native functional properties. RSC Adv 2016;6:44932-43.
4. Lieleg O, Lieleg C, Bloom J, Buck CB, Ribbeck K. Mucin biopolymers as broad-spectrum antiviral agents. Biomacromolecules 2012;13:1724-32.
5. Kočevar-Nared J, Kristl J, Šmid-Korbar J. Comparative rheological investigation of crude gastric mucin and natural gastric mucus. Biomaterials 1997;18:677-81.

6. Celli JP, Turner BS, Afdhal NH, et al. Rheology of gastric mucin exhibits a pH-dependent sol-gel transition. Biomacromolecules 2007;8:1580-6.
7. Lai SK, Wang YY, Wirtz D, Hanes J. Micro- and macrorheology of mucus. Advanced drug delivery reviews 2009;61:86-100.

SUPPLEMENTARY FIGURES

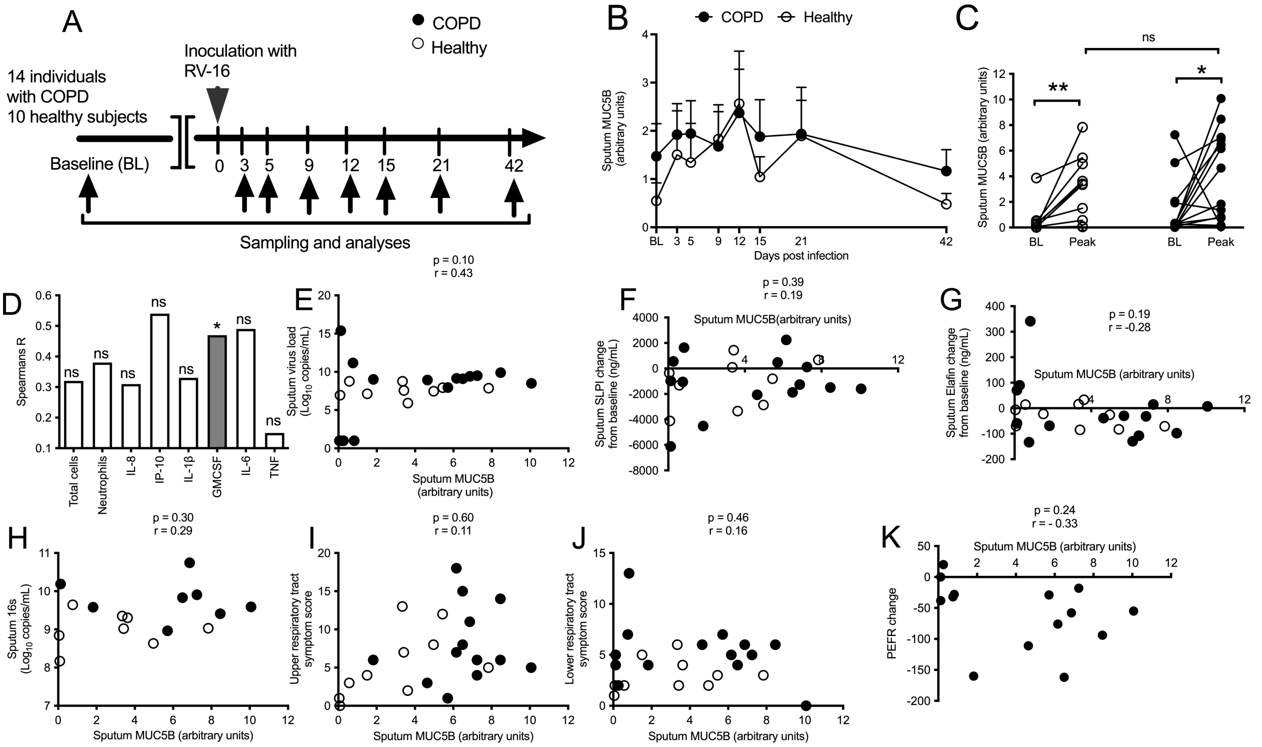

Supplementary Figure 1: Airway MUC5B expression during experimental rhinovirus infection in COPD and healthy subjects. (A) Experimental outline. 14 subjects with COPD and 10 healthy control volunteers underwent sampling and analysis at baseline (~ 14d before RV-A16 inoculation) and at the indicated timepoints after RV-A16 infection. (B) Sputum MUC5B concentrations in COPD and healthy subjects at baseline, and after RV-A16 infection, measured over time. (C) Comparison of baseline and peak (i.e. the maximal concentration of MUC5B detected during the infection for each individual) concentrations of MUC5B and between subjects with COPD and healthy subjects. (D) Correlation of peak sputum MUC5B concentrations with inflammatory cell numbers and concentrations of cytokines in sputum, *$P<0.05$.* Correlation between peak sputum MUC5B and (E) Sputum virus loads. (F) Change from baseline concentrations of SLPI and (G) Change from baseline concentrations of elafin; Correlation of peak sputum MUC5B with (H) bacterial loads assessed by 16S quantitative PCR; (I) Upper respiratory tract symptom scores. (J)Lower respiratory tract symptom scores and (K) Peak expiratory flow (PEF) change from baseline. In (C) individual datapoints are shown and data analysed by Wilcoxon matched-pairs signed rank test for baseline vs peak and Mann Whitney U test for comparison of peak values between COPD and healthy subjects. *$P<0.05$ **$P<0.01$. In (D-K), correlation analysis used was nonparametric (Spearman's) performed on healthy volunteers and individuals with COPD pooled into a single group. In (H) 16S qPCR was not measured in all patients due to lack of sample availability so data only shown where measured. Ns=not significant.

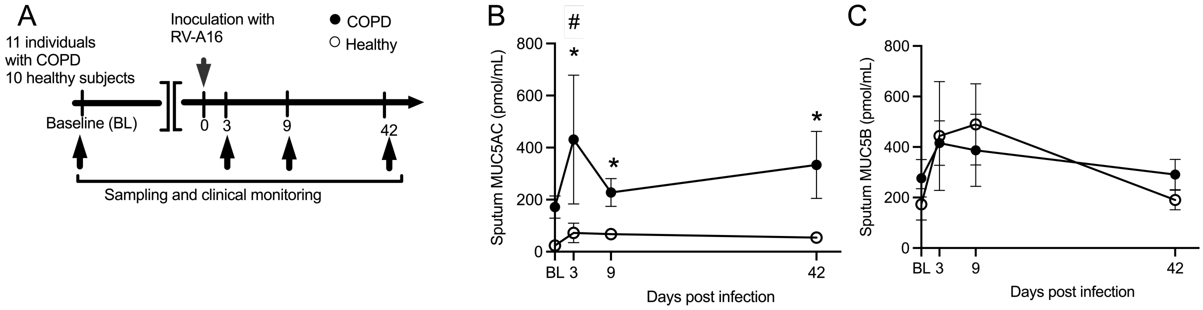

Supplementary Figure 2: Airway mucin expression measured by mass spectrometry during experimental rhinovirus infection in COPD and healthy subjects. (A) Experimental outline. 11 subjects with COPD and 10 healthy control volunteers underwent sampling and mass spectrometry analysis of sputum at baseline (~ 14d before RV-A16 inoculation) and at the indicated timepoints after RV-A16 infection. (B) Sputum MUC5AC and (C) Sputum MUC5B concentrations in COPD and healthy subjects at baseline, and after RV-A16 infection, measured over time. In (B)**P<0.05* (comparison COPD versus healthy) *#P*<0.05 (comparison of day 3 versus baseline in COPD patients).

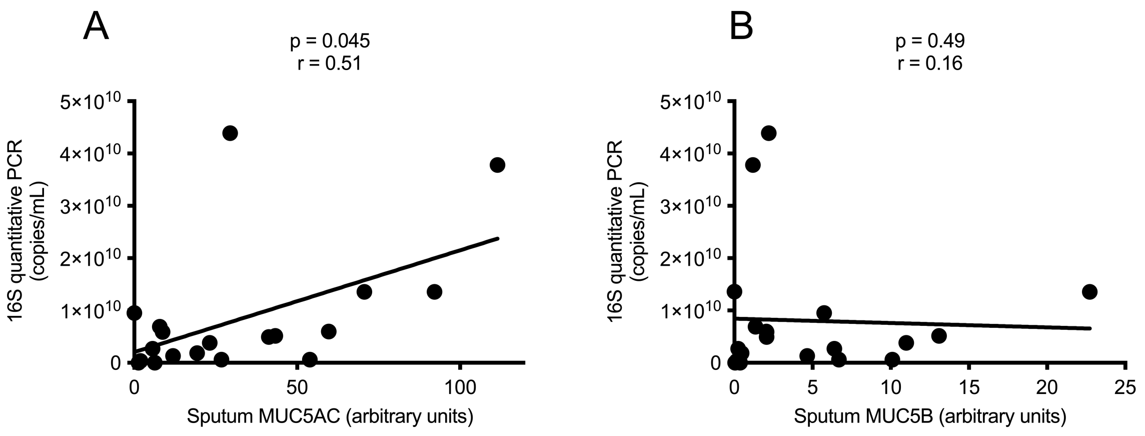

Supplementary Figure 3: Correlation of sputum mucins with bacterial loads during naturally occurring virus-induced COPD exacerbations. (a) sputum MUC5AC and (b) sputum MUC5B correlation with 16S qPCR copies measured at 2 weeks following presentation with virus positive exacerbation in patients with COPD. Correlation analysis used was nonparametric (Spearman's correlation).

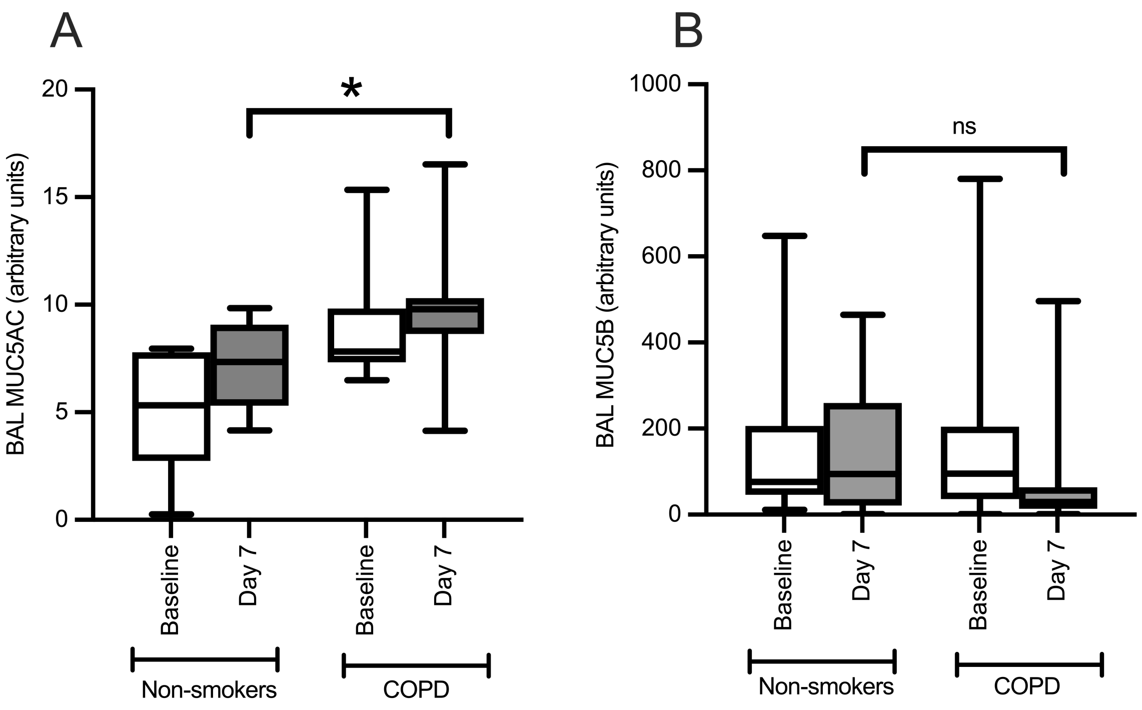

Supplementary Figure 4: Mucin concentrations in bronchoalveolar lavage during experimental rhinovirus infection in COPD and healthy subjects. (A) MUC5AC and (B) MUC5B concentrations in bronchoalveolar lavage (BAL) samples at baseline and 7 days following experimental rhinovirus infection were measured by ELISA in 14 COPD and 10 healthy subjects. Box and whisker plots show median (*line within box*), interquartile range (*box*) and minimum to maximum (*whiskers*) and data analysed by Mann Whitney U test. . **P*<0.05. ns=non-significant.

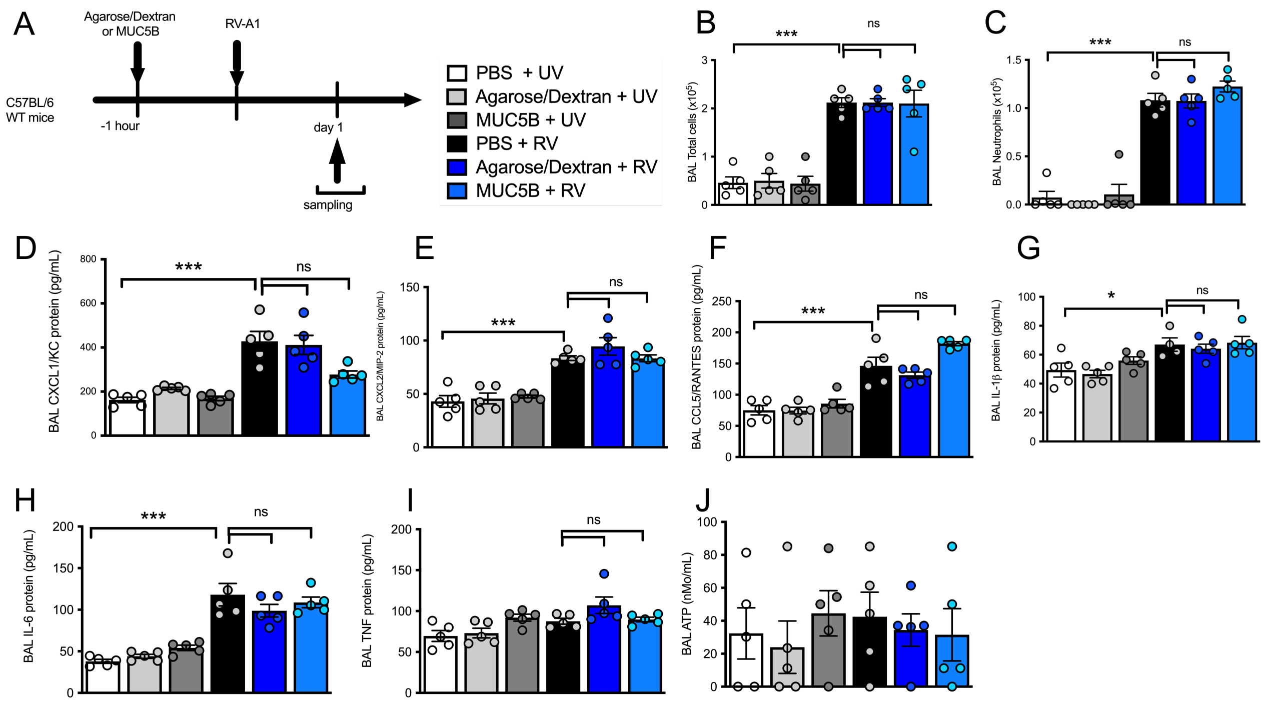

Supplementary Figure 5: No effect of exogenous MUC5B or control polymer of agarose/dextran solution on rhinovirus-induced airway inflammation. (A) Experimental outline. C57BL/6 mice were treated intranasally with purified MUC5B protein or control polymer solution of agarose/dextran and additionally intranasally infected with RV-A1 or UV inactivated RV-A1. (B) Bronchoalveolar lavage (BAL) total cells and (C) BAL neutrophils at day 1 post-infection. BAL concentrations of (D) CXCL1/KC (E) CXCL2/MIP-2 (F) CCL5/RANTES (G)IL-1β (H) IL-6 (I) TNF and (J) ATP at day 1 post-infection. Data in (B-J) expressed as mean (+/-S.E.M) of five mice per treatment group, representative of two independent experiments. Data analysed by one-way ANOVA with Bonferroni's post-test. **P<0.05*; ***P<0.01*; ****P<0.001*. ns=not significant

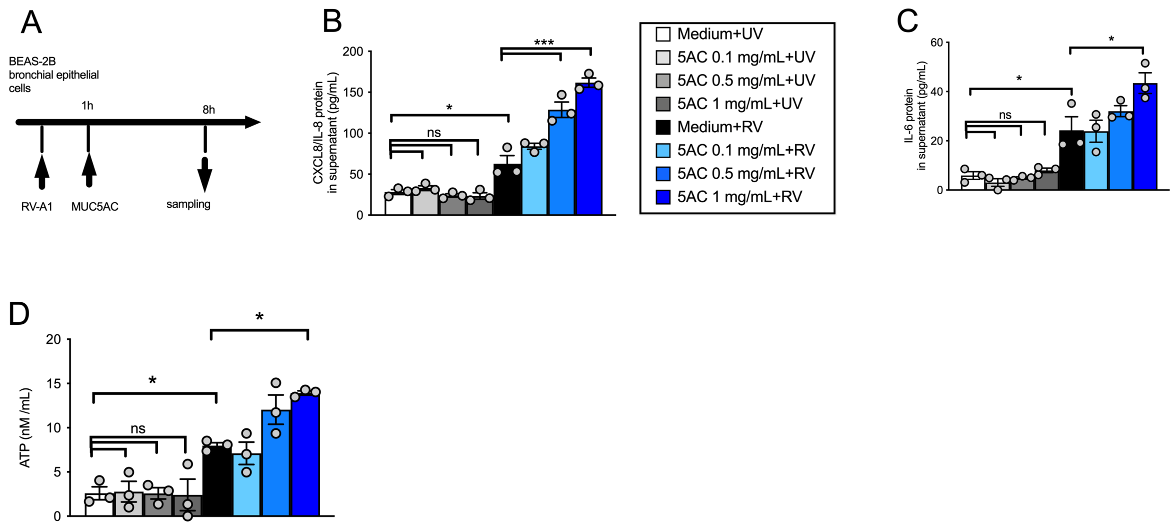

Supplementary Figure 6: Exogenous MUC5AC protein administration augments inflammation and ATP production in human bronchial epithelial cells (A) Experimental outline. BEAS2B bronchial epithelial cells were infected with RV-A1 or UV inactivated RV-A1 and additionally treated with MUC5AC protein. (B) IL-6 and (C) CXCL8/IL-8 protein and (D) ATP concentrations in cell supernatants. Data expressed as median (+/- IQR) of three independent experiments and analysed by Kruskall Wallis test with Dunn's post-test. **P*<0.05 ****P*<0.001. ns = non-significant.

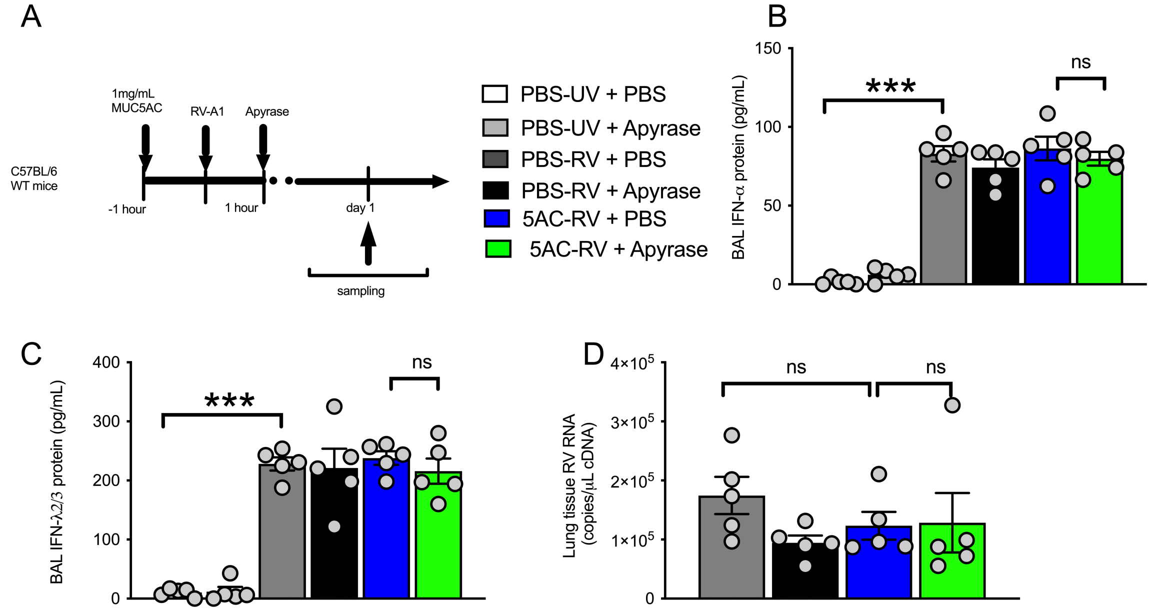

Supplementary Figure 7: No effect of neutralization of airway ATP levels on anti-viral immunity or virus control. (A) Experimental outline. C57BL/6 mice were treated intranasally with purified MUC5AC protein or PBS control, infected with RV-A1 or UV inactivated RV-A1 and additionally treated with intranasal apyrase or vehicle control. (B) Bronchoalveolar lavage (BAL) concentrations of IFN-α and (C) BAL concentrations IFN-λ2/3 proteins at day 1 post-infection. (D) Lung tissue rhinovirus RNA copies at dat 1 post-infection. All data expressed as mean (+/-S.E.M) of five mice per treatment group, representative of two independent experiments. Data analysed by one or two-way ANOVA with Bonferroni's post-test. ns = non-significant. ***P<0.001.

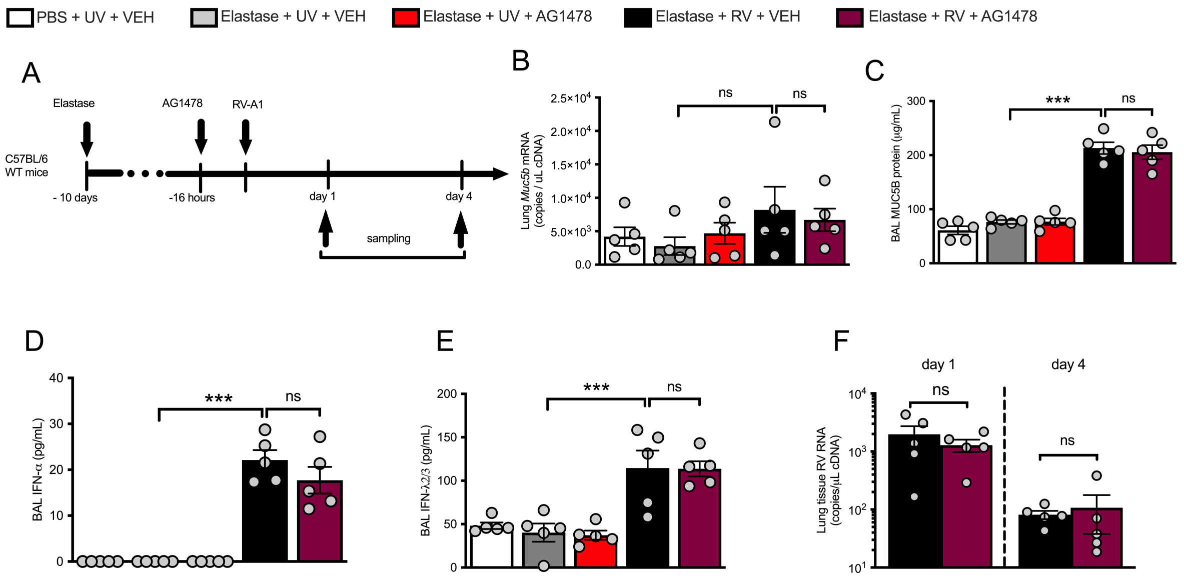

Supplementary Figure 8: No effect of the EGFR inhibitor AG1478 on MUC5B, anti-viral immunity and virus control in a mouse model of rhinovirus-induced COPD exacerbation. (A) Experimental outline. C57BL/6 mice were treated intranasally with elastase or PBS control and additionally treated intraperitoneally with 50mg/kg of EGFR inhibitor AG1478, prior to challenge with rhinovirus (RV)-A1 or UV-inactivated RV-A1 (UV). (B) Lung *Muc5b* mRNA and (C) BAL Muc5B protein concentrations measured at day 1 post-infection. (D) IFN-α and (E) IFN-λ2/3 protein concentrations meaasured at day 1 post-infection. (F) Lung tissue rhinovirus RNA copies. All data expressed as mean (+/-S.E.M) of five mice per treatment group, representative of two independent experiments. Data analysed by one or two-way ANOVA. ns = not significant. ***P<0.001.
